# Global update on the susceptibilities of influenza viruses to neuraminidase inhibitors and the cap-dependent endonuclease inhibitor baloxavir, 2020–2023

**DOI:** 10.1016/j.antiviral.2025.106217

**Published:** 2025-09

**Authors:** Saira Hussain, Adam Meijer, Elena A. Govorkova, Clyde Dapat, Larisa V. Gubareva, Ian G. Barr, Sook Kwan Brown, Rod S. Daniels, Seiichiro Fujisaki, Monica Galiano, Weijuan Huang, Rebecca J. Kondor, Angie Lackenby, Nicola Lewis, Janice Lo, Ha T. Nguyen, Mira C. Patel, Dmitriy Pereyaslov, Aine Rattigan, Magdi Samaan, Dayan Wang, Richard J. Webby, Hui-Ling Yen, Wenqing Zhang, Emi Takashita

**Affiliations:** a WHO Collaborating Centre for Reference and Research on Influenza, Peter Doherty Institute for Infection and Immunity, 792 Elizabeth Street, Melbourne, Victoria, 3000, Australia; b National Institute for Public Health and the Environment, PO Box 1, 3720, BA, Bilthoven, the Netherlands; c WHO Collaborating Centre for Studies on the Ecology of Influenza in Animals, St. Jude Children's Research Hospital, Memphis, TN, 38105-3678, USA; d WHO Collaborating Centre for Surveillance, Epidemiology and Control of Influenza, Centers for Disease Control and Prevention, 1600 Clifton RD NE, MS H17-5, Atlanta, GA, 30329, USA; e WHO Collaborating Centre for Reference and Research on Influenza, The Francis Crick Institute, Worldwide Influenza Centre, 1 Midland Road, London, NW1 1AT, United Kingdom; f WHO Collaborating Centre for Reference and Research on Influenza, National Institute of Infectious Diseases, Gakuen 4-7-1, Musashimurayama, Tokyo, 208-0011, Japan; g WHO Collaborating Centre for Reference and Research on Influenza, National Institute for Viral Disease Control and Prevention, China CDC, Beijing, China; h National Infection Service, Public Health England, London, NW9 5HT, United Kingdom; i Public Health Laboratory Centre, 382 Nam Cheong Street, Hong Kong Special Administrative Region of China; j Global Influenza Programme, World Health Organization, Avenue Appia 20, Geneva 27, 1211, Switzerland; k School of Public Health, Li Ka Shing Faculty of Medicine, The University of Hong Kong, Hong Kong Special Administrative Region of China

**Keywords:** Influenza, Antiviral, Neuraminidase inhibitor, Polymerase inhibitor, Baloxavir, Reduced susceptibility

## Abstract

Antiviral susceptibility of influenza viruses is monitored by the World Health Organization Global Influenza Surveillance and Response System. This study describes a global analysis of the susceptibility of influenza viruses to neuraminidase (NA) inhibitors (NAIs, oseltamivir, zanamivir, peramivir, laninamivir) and the cap-dependent endonuclease inhibitor (CENI, baloxavir) for three periods (May to May for 2020–2021, 2021–2022 and 2022–2023). In particular, global influenza activity declined significantly in 2020–2021 and 2021–2022 when compared to the pre-pandemic period of COVID-19. Combined phenotypic and NA sequence-based analysis revealed that the global frequency of seasonal influenza viruses with reduced or highly reduced inhibition (RI/HRI) by NAIs remained low, 0.09% (2/2224), 0.12% (27/23465) and 0.23% (124/53917) for 2020–2021, 2021–2022 and 2022–2023, respectively. As in previous years, NA-H275Y in A(H1N1)pdm09 viruses was the most frequent substitution causing HRI by oseltamivir and peramivir. Sequence-based analysis of polymerase acidic (PA) protein supplemented with phenotypic testing revealed low global frequencies of seasonal influenza viruses with reduced susceptibility (RS) to baloxavir, 0.07% (1/1376), 0.05% (9/18380) and 0.12% (48/39945) for 2020–2021, 2021–2022 and 2022–2023, respectively; commonly associated substitutions were PA-I38T/M/L. In Japan, the rate was 3.3% (16/488) during 2022–2023, with 11 A(H3N2) viruses having PA-I38T/M substitutions. For zoonotic viruses, 2.7% (3/111) contained substitutions, one each NA-H275Y, NA-S247N and NA-N295S, associated with RI/HRI NAI phenotypes, and none contained PA substitutions associated with RS to baloxavir. In conclusion, the great majority of seasonal and zoonotic influenza viruses remained susceptible to NAIs and CENI baloxavir.

## Highlights

• Antiviral susceptibility to NA inhibitors and PA inhibitor baloxavir was determined for seasonal and zoonotic influenza viruses circulating globally during 2020–2023.
• Low global frequencies (0.1–0.2 %) of seasonal influenza viruses with reduced or highly reduced inhibition by NAI inhibitors were observed as in previous years.
• Low global frequencies of seasonal influenza viruses (∼0.1 %) with reduced susceptibility to baloxavir were observed, with the rate in Japan elevated (3.3 %) in 2022–2023, as has been seen previously.
• For zoonotic viruses, 2.7 % contained genetic markers associated with reduced or highly reduced inhibition to NA inhibitors and none contained markers associated with reduced susceptibility for baloxavir.
• For the treatment of influenza, NA inhibitors and baloxavir remain suitable.

## 1 Introduction

Antiviral treatment and prophylaxis are important interventions to minimize the morbidity and mortality of seasonal, zoonotic, and pandemic influenza. Antivirals available for the treatment of influenza belong to two classes of direct-acting drugs: neuraminidase (NA) inhibitors (NAIs) targeting NA glycoprotein and the cap-dependent endonuclease inhibitor (CENI) targeting polymerase acidic (PA) protein (Jones et al., 2023). Four NAIs are: oral oseltamivir, inhaled or intravenous zanamivir, intravenous peramivir, and inhaled laninamivir (approved only in Japan), with oseltamivir being the most widely used. A single CENI, oral baloxavir marboxil (baloxavir), was approved in Japan and the United States in 2018 and is now approved in more than 70 countries (Beigel and Hayden, 2021; Ison et al., 2021; Roche, 2022).

The emergence of viruses with reduced susceptibility to antivirals poses a continuous risk to limit their efficacy. More than 90% of former seasonal A(H1N1) viruses during 2007–2009 showed highly reduced inhibition (HRI) by oseltamivir and peramivir associated with the NA-H275Y substitution (Dharan et al., 2009; Hurt et al., 2009a; McKimm-Breschkin, 2013; Meijer et al., 2009). Consequently, antiviral susceptibility monitoring has become an integral part of influenza virologic surveillance conducted by laboratories participating in the World Health Organization (WHO) Global Influenza Surveillance and Response System (GISRS). In recent years, the overall frequency of viruses showing reduced inhibition (RI)/HRI by NAIs or reduced susceptibility (RS) to baloxavir has been low (<2%) (Govorkova et al., 2022; Gubareva et al., 2017; Hurt et al., 2016; Lackenby et al., 2018; Meijer et al., 2014; Takashita et al., 2015, 2020b).

This is the eighth WHO-Antiviral Working Group (WHO-AVWG) review of antiviral susceptibility. Data was collated from five WHO Collaborating Centres (WHO CCs) for Reference and Research on Influenza, one WHO CC for Studies on the Ecology of Influenza in Animals, one WHO CC for Studies on Influenza at the Animal-human Interface (VECTOR) and National Influenza Centres (NICs); the data from VECTOR and NICs and other laboratories was sequence-based analysis only as retrieved from GISAID. It includes influenza antiviral susceptibility data for three consecutive periods spanning May (week 21) to May (week 20) of the following year for 2020–2021, 2021–2022 and 2022–2023, as used in previous global reports (thereby covering Southern and subsequent Northern Hemisphere influenza seasons).

During the COVID-19 pandemic, influenza virus circulation decreased significantly compared to previous seasons (Takashita et al., 2023b). Global influenza circulation was still low in 2021 and increased in the 2022–2023 season as compared with 2018–2019 and 2019–2020 seasons based on the global web-based tool for influenza virologic surveillance (FluNet, https://www.who.int/tools/flunet). It is also important to note that influenza B/Yamagata-lineage virus circulation has not been confirmed since March 2020 (Caini et al., 2024).

NICs conduct initial analysis of influenza virus-positive clinical specimens collected in their respective countries. They then share clinical specimens and/or virus isolates representative of each influenza type and subtype lineage with WHO CCs. At the CCs viruses are propagated in MDCK, MDCK-SIAT1, or hCK cells for phenotypic drug susceptibility testing.

With the advances in next-generation sequencing (NGS) technologies, there is increased emphasis on establishing sequence-based virologic surveillance, particularly since the COVID-19 pandemic. GISRS laboratories share their virus sequences via the Global Initiative on Sharing All Influenza Data (GISAID), allowing viruses to be screened for likely reduced antiviral susceptibility by identification of known or suspected amino acid substitutions (markers) in the protein targeted by the drug. When virus isolates are available this information is used to select marker-containing viruses for phenotypic testing compared with subsets of viruses without markers. Details on the different Next generation sequencing protocols used by WHO CCs are in Table S1.

Phenotypic assays for the determination of 50% inhibitory concentrations (IC_50_) for NAIs are fluorescence-based or chemiluminescence-based. Most GISRS laboratories performing phenotypic NAI testing use versions of fluorescence-based NA inhibition assays with MUNANA (4-(methylumbelliferyl)-N-acetylneuraminic acid) as substrate (Meijer et al., 2014; Potier et al., 1979). Details on the different protocols used by WHO CCs are in Table S2 and are summarized by the WHO-AVWG (https://www.who.int/teams/global-influenza-programme/laboratory-network/quality-assurance/antiviral-susceptibility-influenza/neuraminidase-inhibitor). For routine monitoring of antiviral susceptibility, IC_50_ values for viruses are compared to a reference IC_50_ value, e.g., a median IC_50_ of viruses of the respective type/subtype/lineage known to be susceptible to the NAIs. Antiviral susceptibility of influenza viruses is classified as normal inhibition (NI) (<10-fold reduction in inhibition for type A viruses; <5-fold for type B viruses), RI (10- to 100-fold for type A viruses; 5- to 50-fold for type B viruses) and HRI (>100-fold for type A viruses; >50-fold for type B viruses) (WHO, 2012). Amino acid substitutions that have been associated with RI/HRI phenotypes are summarized by the WHO-AVWG (NA marker tables: https://www.who.int/teams/global-influenza-programme/laboratory-network/quality-assurance/antiviral-susceptibility-influenza/neuraminidase-inhibitor).

For phenotypic assessment of susceptibility to CENI baloxavir, influenza virus replication in cell culture in the presence of the drug is monitored. Four assays are currently used by the WHO CCs, high-content imaging neutralization test (HINT), influenza replication inhibition neuraminidase-based assay (IRINA), focus reduction assay (FRA), and plaque reduction assay (PRA) (Gubareva et al., 2019; Patel et al., 2022; Takashita et al., 2018). These assays yield different effective concentration (EC_50_) values for a given virus, but the fold changes compared to controls are similar (Takashita et al., 2020b). Details on different assays used by WHO CCs are in Table S3 and are summarized by the WHO-AVWG (https://www.who.int/teams/global-influenza-programme/laboratory-network/quality-assurance/antiviral-susceptibility-influenza/polymerase-acidic-protein-inhibitor). Currently, there are no criteria for defining resistance or RS to baloxavir. An arbitrary threshold (cut-off) of a >3-fold increase in EC_50_ compared to reference EC_50_ (median) is used for reporting viruses with RS to baloxavir (Govorkova et al., 2022; Gubareva et al., 2019; Takashita et al., 2020b). This cut-off should capture >95% of viruses with RS to baloxavir (Ince et al., 2020). RS to baloxavir is most commonly associated with amino acid substitution at residue 38 in the PA protein, with PA-I38T being particularly common (Imai et al., 2020; Omoto et al., 2018), and frequencies of baloxavir treatment–emergent variants differing between virus types and subtypes (Hayden et al., 2018; Ince et al., 2020; Ison et al., 2020; Uehara et al., 2020). PA substitutions associated with RS are summarized by the WHO-AVWG (PA marker table: https://www.who.int/teams/global-influenza-programme/laboratory-network/quality-assurance/antiviral-susceptibility-influenza/polymerase-acidic-protein-inhibitor).

This report summarizes combined sequence-based and phenotypic data for antiviral susceptibility monitoring to NAIs and CENI baloxavir from WHO CCs, in addition to sequence-based data provided by NICs for seasonal and zoonotic influenza viruses (which have the potential to cause pandemics) isolated from humans in different countries and retrieved from GISAID by the WHO CCs.

## 2 Neuraminidase inhibitors (NAIs)

### 2.1 Analysis of phenotypic and sequence-based NAI susceptibility data from WHO CCs

**Fig. 1:**
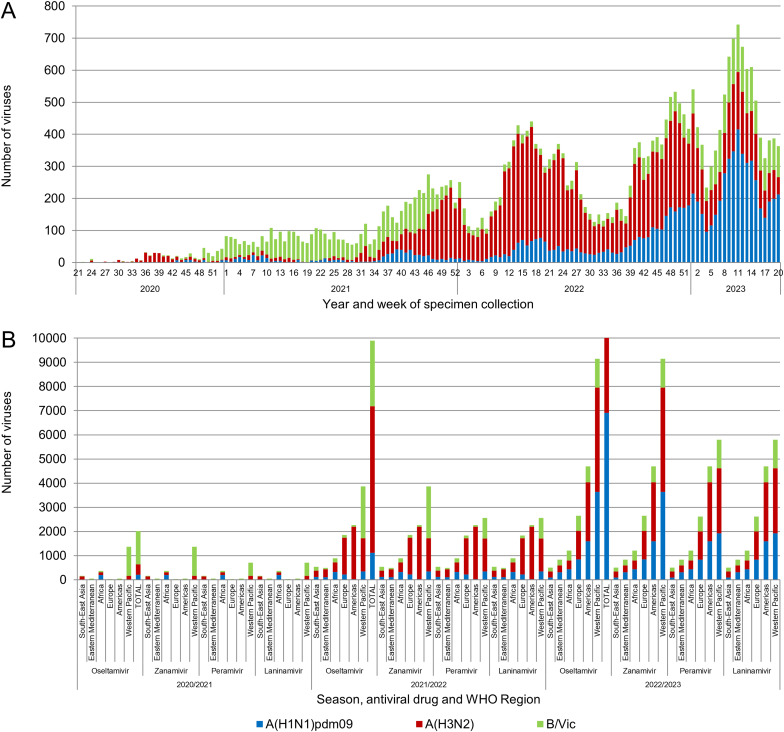
Influenza viruses collected and assessed for NAI susceptibility during the 2020–2021, 2021–2022 and 2022–2023 periods by the WHO CCs. For each period viruses were collected from patients at week 21 through week 20 of the following year. (A) The number of viruses collected by year and week of specimen collection and virus type/subtype or lineage for specimens assessed in the three consecutive periods. Typically, week 21 to week 39 of a year covers the Southern Hemisphere influenza season, while week 40 of a year to week 20 of the following year covers the Northern Hemisphere influenza season. (B) The number of viruses assessed for susceptibility to the four NAIs using NA inhibition assays and/or sequence-based analysis by WHO Region for the three consecutive periods. For 2021–2022 and 2022–2023, two reassortant A(H1N2) viruses were counted among the A(H3N2) viruses because of assessment of N2 NA for NAI susceptibility but these are not shown on the graph.

**Fig. 2:**
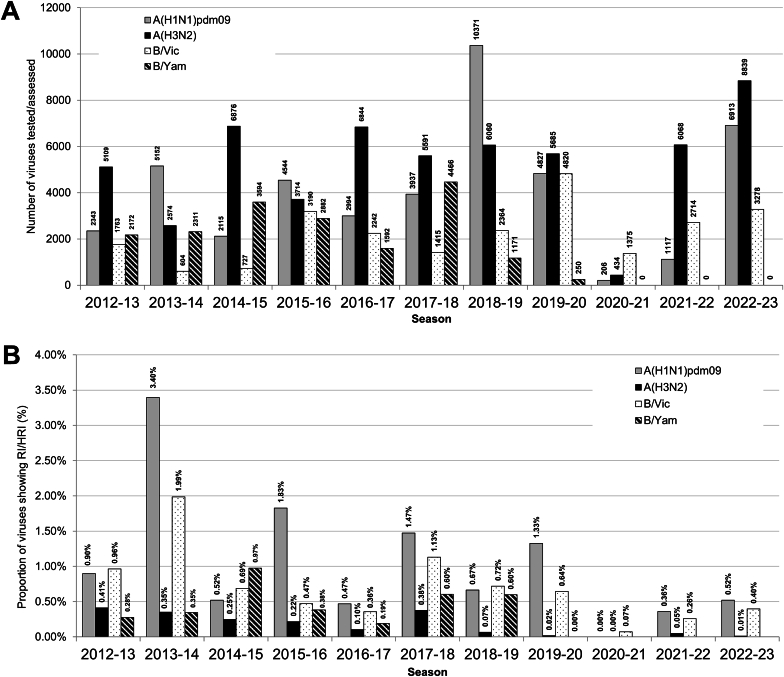
Comparison of NAI susceptibility surveillance over 11 periods for phenotypic and/or sequence-based analysis data generated by WHO CCs. (A) Number of viruses tested. For the 2012–2018 periods testing was reported based on NA inhibition assays only. For the subsequent five periods, results of assessment by NA inhibition assays and/or sequence-based analysis were included. (B) The proportion of viruses showing RI/HRI by one or more NAIs over the 2012–2023 periods. Data were compiled from the global studies of viruses analyzed by WHO CCs during the 2012–2013 (Meijer et al., 2014), 2013–2014 (Takashita et al., 2015), 2014–2015 (Hurt et al., 2016), 2015–2016 (Gubareva et al., 2017), 2016–2017 (Lackenby et al., 2018), 2017–2018 (Takashita et al., 2020b), 2018–2020 (Govorkova et al., 2022), and 2020–2023 (current study) periods. For 2021–2022 and 2022–2023, reassortant A(H1N2) viruses were counted among the A(H3N2) viruses because of assessment of N2 NA for NAI susceptibility. Based on sequence-based analysis, viruses were considered RI or HRI when the NA contained an amino acid substitution for which the NA marker table posted on the WHO website and/or current testing data showed an IC_50_ fold change of >10 (for A viruses) or >5 (for B viruses) compared to period median IC_50_ values by subtype/lineage, WHO-CC and used method. When a particular amino acid substitution listed as RI or HRI at the WHO website was phenotypically confirmed NI in the genetic background of current viruses, all with such an NA amino acid substitution were considered as displaying NI. These were A(H3N2) viruses with NA-N329K/R or NA-S331R substitutions and B/Victoria lineage viruses with NA-K360E substitution.

During the three consecutive periods, totals of 2015, 9899 and 19030 viruses, respectively, were assessed for NAI susceptibility by five WHO CCs (Atlanta, London, Tokyo, Melbourne and China) using either phenotypic, sequence-based, or both methods (Fig. 1, Fig. 2). These were seasonal influenza A(H1N1)pdm09, A(H3N2) and B/Victoria-lineage viruses (only one A(H1N2) virus in each period 2021–2022 and 2022–2023). Of these respective total numbers 1447 in 2020–2021, 5287 in 2021–2022 and 10558 in 2022–2023 were phenotypically tested by an NA inhibition assay. All these viruses were tested for susceptibility to oseltamivir and zanamivir, except for two viruses in 2021–2022, which were tested only with zanamivir. Three WHO CCs (Atlanta, Melbourne and Tokyo) also tested viruses for susceptibility to peramivir and laninamivir. The remaining viruses were screened for NA markers. The number of influenza viruses by type/subtype or lineage collected and assessed for NAI susceptibility during the three consecutive periods by the WHO CCs is presented in Fig. 1. Most viruses originated from the WHO regions of the Western Pacific (WPR with three CCs; 67.8%, 39.0% and 48.1% for the three consecutive periods) and the Americas (AMR; 22.9% for 2021–2022 and 24.7% for 2022–2023, albeit the percentage being very low (2.6%) in 2020–2021). Only 29.6%, 38.1% and 27.2% for the three consecutive periods were from the African (AFR), Eastern Mediterranean (EMR), European (EUR) and Southeast Asian (SEAR) regions. Totals of 11728, 402482 and 1167075 influenza viruses were detected globally and reported to FluNet for the three consecutive periods. Therefore, the viruses analyzed for NAI susceptibility by the WHO CCs in this study represent 17.2%, 2.5% and 1.6% of reported global influenza detections for the three consecutive periods.

For 2020–2021, influenza B/Victoria-lineage viruses were the most prevalent among the viruses assessed (1375; 68.2%), followed by A(H3N2) (434; 21.5%) and A(H1N1)pdm09 (206, 10.2%) viruses. For 2021–2022, A(H3N2) viruses were the most prevalent among the viruses assessed (6067; 61.3%), followed by B/Victoria-lineage (2714; 27.4%) and A(H1N1)pdm09 (1117; 11.3%) viruses. For 2022–2023, A(H3N2) viruses were again the most prevalent among the viruses assessed (8838; 46.4%), followed by A(H1N1)pdm09 (6913; 36.3%) and B/Victoria lineage (3278; 17.2%) viruses.

Testing in the NA inhibition assay identified 1/1447 viruses from 2020–2021, 7/5287 viruses from 2021–2022, and 37/10558 from 2022–2023 with RI/HRI. Based on NA sequence analysis small numbers of additional viruses carrying NA markers were identified: zero, seven and 13, respectively, for the three consecutive periods. Overall, 1/2015 (0.05%), 14/9899 (0.14%), and 50/19030 (0.26%) viruses from 2020–2021, 2021–2022 and 2022–2023, respectively, carried markers for RI/HRI by at least one NAI. These frequencies are lower than the corresponding result (0.5%) for 2018–2020 periods (Govorkova et al., 2022).

### 2.2 A(H1N1)pdm09 viruses showing RI/HRI

Increases in the frequencies of viruses exhibiting RI/HRI by at least one NAI were seen across the three consecutive periods: 0/206 (0.00%), 4/1117 (0.36%), and 36/6913 (0.52%) (Fig. 2).

**Fig. 3:**
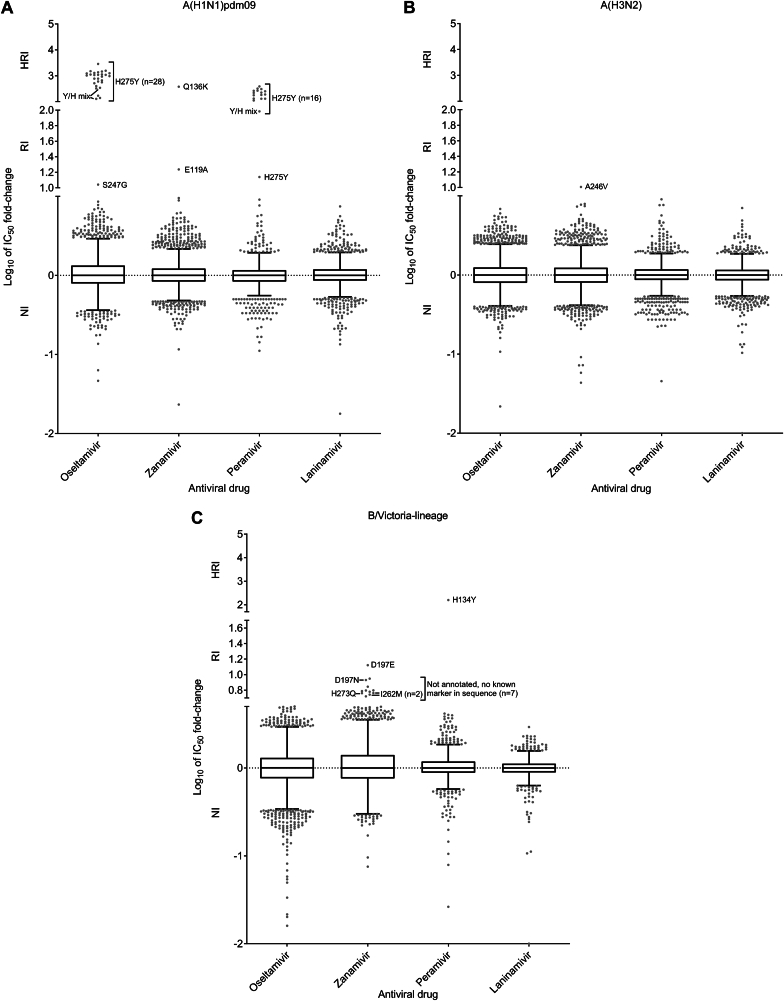
Column-scatter plots of log-transformed 50% inhibitory concentration (IC_50_) fold-change values for NAIs. Overall, 1447/2015, 5287/9899 and 10558/19030 viruses assessed for NAI susceptibility were tested phenotypically for one or more NAIs for 2020–2021, 2021–2022 and 2022–2023 periods, respectively. Data are presented by virus subtype or lineage [(A) A(H1N1)pdm09; (B) A(H3N2); and (C) B/Victoria-lineage] with NAI (labelled on the x-axis: oseltamivir, zanamivir, peramivir, and laninamivir) and log-transformed IC_50_ fold-changes compared to the period median on the y-axis. The boxes indicate the 25th–75th percentiles, and the whiskers stretch to the lowest and highest values within 1.5 times the interquartile region (IQR) value from both the 25th and 75th percentile values, respectively (Tukey's definition). The y-axes have been split into three compartments according to the thresholds recommended by the WHO-AVWG for NI (<10-fold for type A viruses; <5-fold for type B viruses), RI (10- to 100-fold for type A viruses; 5- to 50-fold for type B viruses), and HRI (>100-fold for type A viruses; >50-fold for type B viruses). NA amino acid substitutions are shown for viruses displaying RI or HRI phenotypes that have been sequenced. For several viruses with RI phenotype that have been sequenced, no amino acid substitution marker associated with this phenotype was identified. For another number of viruses with RI phenotype no sequence was available for evaluation. Viruses showing NI but carrying amino acid substitutions previously associated with RI or HRI by one or more other NAI are not indicated in the figure. These were A(H3N2) viruses with NA-N329K/R or NA-S331R substitutions and B/Victoria lineage viruses with NA-K360E substitution. Amino acid position numbering is specific to A subtype and B type. Most viruses were tested for susceptibility to oseltamivir and zanamivir; only a subset was tested for susceptibility to peramivir and laninamivir.

Most viruses with an RI/HRI phenotype contained the NA-H275Y substitution (33/40, 82.5%) and showed the expected increases in IC_50_ for oseltamivir and peramivir (Table 1 and S4, Fig. 3A). In addition, two viruses had an NA-H275Y/H mixture and showed elevated IC_50_ for oseltamivir and peramivir. These NA-H275Y variants were collected in 11 countries. Of these 35 viruses, for 16 with NA-H275Y or NA-H275Y/H, the NA-H275Y substitution was confirmed in the corresponding clinical specimens; another virus showed NA-D199G (majority) and NA-H275Y (minority) mixture in the clinical specimen but only NA-H275Y substitution was identified in the isolate; no clinical specimens were available or sequenced for the remaining 18 viruses. Of the five patients with available clinical history, all were hospitalized. Antiviral treatment history was available for only nine patients: two were treated with oseltamivir; one with zanamivir (direct communication with the hospital indicated this patient was first treated with oseltamivir and results showed the patient acquired NA-H275Y during this period); six patients had not received an NAI before specimen collection. Immunocompromised status was reported for three patients who shed viruses with NA-H275Y substitution.

**Table 1:** WHO CC data: Virus and patient characteristics for influenza type A viruses (n = 44) showing (tested phenotypically) and/or associated (sequence-based analysis) with RI/HRI by NAIs.^a^

Virus (n)	n (sequence-based analysis)	IC_50_ fold-change compared to reference median IC_50_ values^b^				NA substitution^c^		Patient setting	Antiviral treatment	Immunocompromised
		Oseltamivir	Zanamivir	Peramivir	Laninamivir	Virus isolate	Clinical specimen			
										
**A(H1N1)pdm09** (40 out of 8236)	33 (7)	**139–2846**	0.47–1.92	**14–386**	0.83–4.88	H275Y (31), Not available (2)	H275Y (16), D199G/H275Y (1), No data (3), Not available (10), Not sequenced (3)	Hospital (4), Unknown (29)	Yes, oseltamivir (2), No (6), Unknown (25)	Yes (2), No (2), Unknown (29)
	2	**128–341**	0.90–1.07	**96**	2.05	H275Y/H	Not available (1), Not sequenced (1)	Hospital (1), Unknown (1)	Yes, zanamivir (1), Unknown (1)	Yes (1), Unknown (1)
	2 (2)	n/t^d^	n/t	n/t	n/t	I223T	I223T (1), No data (1)	Community (1), Unknown (1)	No (1), Unknown (1)	No (1), Unknown (1)
	1	6.34	**17.24**	n/t	n/t	E119A	Not available	Unknown	Unknown	Unknown
	1	0.49	**375**	n/t	n/t	Q136K	Not available	Unknown	Unknown	Unknown
	1	**11.00**	1.00	1.25	1.14	S247G	S247G	Unknown	Unknown	Unknown
**A(H3N2)** (4 out of 15339)	2 (2)	n/t	n/t	n/t	n/t	E119V	E119V (1), No data (1)	Unknown	Unknown	Unknown
	1	1.86	**10.16**	n/t	n/t	A246V	A246V	Hospital	No	Unknown
	1 (1)	n/t	n/t	n/t	n/t	K249E	K249E	Unknown	Unknown	Unknown

a The number (n) of viruses for which sequence data only were reported is shown in parentheses.

b RI and HRI fold-change values are displayed underlined and in bold typeface. For type A viruses, normal inhibition (NI) is a <10-fold increase in the NAI IC_50_; RI is a 10- to 100-fold increase; and HRI is a >100-fold increase (WHO, 2012).

c Amino acid position numbering is A-subtype specific. NA amino acid substitutions associated with RI/HRI, as listed in the summary table provided by the WHO-AVWG on the WHO website (https://www.who.int/teams/global-influenza-programme/laboratory-network/quality-assurance/antiviral-susceptibility-influenza/neuraminidase-inhibitor) are shown.

d n/t: not tested.

Two viruses with NA-I223T substitution were not tested in NA inhibition assay and the substitution was present in the one clinical specimen available for sequencing (Table 1 and S4). One virus, recovered from a patient with unknown antiviral treatment status (Table 1 and S4), exhibited RI by zanamivir (17.24-fold) conferred by NA-E119A substitution (clinical specimen not available) (Fig. 3A). One virus contained NA-Q136K substitution and showed HRI by zanamivir (Fig. 3A); this substitution has been associated with cell-culture selection and clinical specimen was not available to assess this (Table 1 and S4). One virus had NA-S247G substitution (confirmed in clinical specimen) and showed borderline RI by oseltamivir (11.00-fold) (Fig. 3A).

### 2.3 A(H3N2) viruses showing RI/HRI

As in previous periods, the frequency of A(H3N2) viruses showing RI/HRI by any NAI remained very low (Fig. 2). Over the three consecutive periods, 0/434 (0.00%), 3/6067 (0.05%), and 1/8838 (0.01%) viruses, respectively, exhibited RI by at least one NAI. These frequencies were similar to the corresponding result (0.04%) for 2018–2020 periods (Govorkova et al., 2022).

In 2021–2022 and 2022–2023 periods, two viruses bearing the NA-E119V substitution were detected in Europe, and the substitution was confirmed in the one clinical specimen available for sequencing (Table 1 and S4). These viruses were not phenotypically tested for NAI susceptibility. However, NA-E119V was previously found to be associated with RI/HRI by oseltamivir (NA marker tables). A single A(H3N2) virus bearing NA-K249E (also found in the clinical specimen), previously reported to be associated with RI by oseltamivir (Gubareva et al., 2017), was collected in Oman (without patient setting and antiviral treatment data) during the 2021–2022 period and was not phenotypically tested for NAI susceptibility (Table 1 and S4). Another virus from Lebanon, bearing NA-A246V (also found in the clinical specimen), showed borderline RI by zanamivir (10.16-fold) (Fig. 3B), was collected from a hospitalized patient who had not been NAI treated before specimen collection (Table 1 and S4). NA-A246V had not previously been associated with RI by NAIs in this (N2) subtype but NA-N245Y has been associated with RI by oseltamivir (Govorkova et al., 2022) and deletions at NA-245-248 (Abed et al., 2009; Tamura et al., 2015) and NA-247-250 (Tamura et al., 2015) have been associated with HRI by oseltamivir and NI/RI by zanamivir.

### 2.4 B/Victoria-lineage viruses showing RI/HRI

For B/Victoria-lineage viruses, 1/1375 (0.07%), 7/2714 (0.26%) and 13/3278 (0.40%) viruses assessed over the three consecutive periods exhibited RI/HRI by at least one NAI (Fig. 2). These frequencies are lower than the corresponding result (0.70%) for 2018–2020 periods (Govorkova et al., 2022). Viruses with RI/HRI were collected from 12 countries (Table S4). Six viruses collected in 2022–2023 from Europe showed mild elevation in zanamivir IC_50_s (5.25- to 8.86-fold) (Fig. 3C) and no known NA markers were detected in the isolates or the available clinical specimens (5/6) (Table 2 and S4).

**Table 2:** WHO CC data: Virus and patient characteristics for influenza type B viruses (n = 21) showing (tested phenotypically) and/or associated (sequence-based analysis) with RI/HRI by NAIs.^a^

Virus (n)	n (sequence-based analysis)	IC_50_ fold-change compared to reference median IC_50_ values^b^				NA substitution^c^		Patient setting	Antiviral treatment	Immunocompromised
		Oseltamivir	Zanamivir	Peramivir	Laninamivir	Virus isolate	Clinical specimen			
**B/Victoria-lineage** (21 out of 7367)	6	0.77–1.85	**5.25–8.86**	n/t^d^	n/t	None^e^	None (5), No data (1)	Unknown	Yes, Unknown (1), No (3), Unknown (2)	Unknown
	4 (3)	**5.00**	**8.52**	n/t	n/t	D197N	Not available (1), No data (3)	Hospital (1), Unknown (3)	Unknown	Unknown
	3 (2)	3.76	**13.24**	n/t	n/t	D197E (2), Not available (1)	D197E (1), Not available (1), No data (1)	Hospital (1), Unknown (2)	Unknown	Unknown
	2	1.09–1.76	**5.76–6.01**	n/t	n/t	I262M^f^	No data	Unknown	Unknown	Unknown
	1	2.49	1.04	**158**	0.91	H134Y	None	Unknown	Unknown	Unknown
	1 (1)	n/t	n/t	n/t	n/t	G145E	No data	Unknown	Unknown	Unknown
	1 (1)	n/t	n/t	n/t	n/t	T146K	No data	Unknown	Unknown	Unknown
	1	2.19	**6.25**	n/t	n/t	S244P	No data	Unknown	Unknown	Unknown
	1	0.06	**5.79**	n/t	n/t	H273Q	Not available	Hospital	Unknown	Unknown
	1 (1)	n/t	n/t	n/t	n/t	G407S	No data	Unknown	Unknown	Unknown

a The number (n) of viruses for which sequencing data only were reported is shown in parentheses.

b RI and HRI fold-change values are displayed underlined and in bold typeface. For type B viruses, normal inhibition (NI) is a <5-fold increase in the NAI IC_50_; RI is a 5- to 50-fold increase; and HRI is a >50-fold increase (WHO, 2012).

c Amino acid position numbering is B-type specific. NA amino acid substitutions associated with RI/HRI, as listed in the summary table provided by the WHO-AVWG on the WHO website (https://www.who.int/teams/global-influenza-programme/laboratory-network/quality-assurance/antiviral-susceptibility-influenza/neuraminidase-inhibitor) are shown.

d n/t: not tested.

e None: No known substitution(s) associated with reduced inhibition by NAIs.

f NA-I263M substitution, if accounting for insertion at NA-73L.

The most frequently occurring RI/HRI markers were at residue 197 of NA (Table 2 and S4, Fig. 3C). Substitutions at this position, NA-D197E/G/N/Y have been associated with borderline RI by NAIs (NA marker tables). Four viruses possessed NA-D197N, previously associated with NI/RI by oseltamivir, zanamivir and peramivir; one of these was tested phenotypically and showed RI by oseltamivir and zanamivir (peramivir and laninamivir were not tested). No data was available for the corresponding clinical specimens. One patient had been hospitalized and patient setting was unknown for the other three; antiviral treatment status was unknown for all patients. Three viruses possessed NA-D197E, previously associated with RI by oseltamivir and peramivir (Burnham et al., 2014; Hurt et al., 2006; Monto et al., 2006); one was tested phenotypically and showed RI by zanamivir (13.24-fold) and borderline RI by oseltamivir (3.76-fold). Another was from a clinical specimen, for which an isolate was not available. One patient was hospitalized, and patient setting was unknown for the other two; antiviral treatment status was unknown for all patients.

It is known that certain cell-culture and virus propagation conditions favor the rapid selection of influenza B virus variants with NA substitutions, which present challenges for phenotypic testing (Govorkova et al., 2022). NA substitutions (e.g., NA-H101L, NA-T146I/K/P and NA-D197G) known to occur due to adaptation of influenza B viruses to growth in cell-culture have been reported (Brown et al., 2022; Hurt et al., 2016). Two viruses bearing NA-I262M and one each bearing NA-S244P or NA-H273Q showed borderline RI by zanamivir (5.76- to 6.25-fold) (Fig. 3C). No clinical specimen data was available for these isolates (Table 2 and S4). Antiviral treatment status was unknown for all patients. One virus bearing NA-H134Y showed HRI by peramivir (Fig. 3C), but the substitution was not found in the clinical specimen and no further patient and antiviral treatment data were available (Table 2 and S4). Three viruses bearing NA-G145E, NA-T146K, or NA-G407S were not phenotypically tested for NAI susceptibility, and clinical specimen data, patient setting, and antiviral treatment status were unknown for all patients (Table 2 and S4).

### 2.5 Frequency of NA markers associated with RI/HRI by NAIs in sequence databases

We analyzed NA sequences of viruses that were deposited into GISAID, removing duplicate strain designations (preferring sequence data from the original specimen where possible) that were from specimens collected during the 2020–2021 (1552 viruses), 2021–2022 (21466 viruses) and the 2022–2023 (48375 viruses) periods. According to the strain designation, 1343 sequences from 2020–2021, 7899 sequences from 2021–2022 and 13487 sequences from 2022–2023 belonged to viruses characterized by five WHO CCs for NAI susceptibility. For the remaining 209 viruses [40 A(H1N1)pdm09, 102 A(H3N2), 67 B/Victoria-lineage] from 2020–2021, 13567 viruses [919 A(H1N1)pdm09, 12074 A(H3N2), 574 B/Victoria] from 2021–2022 and 34888 viruses [10471 A(H1N1)pdm09, 17802 A(H3N2), 6615 B/Victoria] from 2022–2023, NA sequences were analyzed for the presence of NA markers. For 2020–2021, one A(H1N1)pdm09 virus [0.48% of the total], for 2021–2022, 13 viruses [0.10% of the total; three A(H1N1)pdm09, five A(H3N2), five B/Victoria-lineage] and for 2022–2023, 74 viruses [0.21% of the total; 45 A(H1N1)pdm09, 16 A(H3N2), 13 B/Victoria-lineage] were identified with NA markers (Table S5). Mutation calling on sequences downloaded from GISAID was performed using Nextclade (version 3.15.1) (Aksamentov et al., 2021).

Of the 11430 A(H1N1)pdm09 sequences analyzed, 49 (0.43%) viruses contained NA markers associated with RI/HRI: of these 32 (65.3%) viruses contained NA-H275Y, two (4.1%) viruses contained NA-D199G, two (4.1%) viruses contained NA-I223M, three (6.1%) viruses contained NA-I223R, four (8.2%) viruses contained NA-I223T and one virus each (12.2% total) contained NA-I223K, NA-I223V + H275Y, NA-Q136R, NA-R152K, NA-S247G or NA-N295S substitutions.

Of the 29978 A(H3N2) sequences analyzed, 21 (0.07%) viruses contained NA markers associated with RI/HRI: eight (38.1%) viruses had NA-K249E, six (28.6%) viruses had NA-E119V, three (14.3%) viruses had NA-D151G, and one virus each (19.0% total) had NA-N142S, NA-N245Y, NA-E276D or NA-R292K substitutions.

Of the 7256 B/Victoria-lineage sequences analyzed, 18 (0.25%) viruses contained NA markers associated with RI/HRI: three (16.7%) viruses had NA-D197N, four (22.2%) viruses showed NA-N151S and one virus each (27.8% total) had NA-D197E, NA-I221L, NA-I221T, NA-I221V or NA-G243S. Two viruses each contained NA-H273Y, NA-G407S or NA-D432N substitutions (33.3% total).

Overall, a combined analysis using phenotypic, sequence-based, or both methods revealed that the global frequency of all seasonal influenza viruses with RI/HRI by NAIs or carrying NA markers associated with RI/HRI phenotypes was low, being 0.09% (2/2224), 0.12% (27/23465) and 0.23% (124/53917) for the three consecutive periods.

## 3 Cap-dependent endonuclease inhibitor

### 3.1 Analysis of phenotypic and sequence-based CENI baloxavir susceptibility data from WHO CCs

**Fig. 4:**
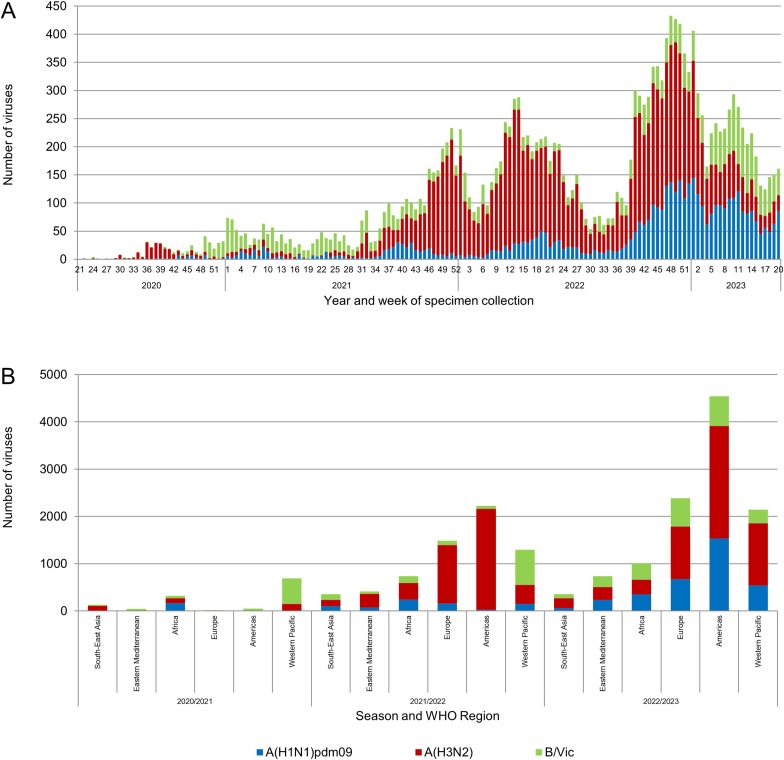
Influenza viruses collected and assessed for baloxavir susceptibility during the 2020–2021, 2021–2022 and 2022–2023 periods by the WHO CCs. For each period viruses were collected from patients at week 21 through week 20 of the following year. (A) The number of viruses collected by year and week of specimen collection and virus type/subtype or lineage for specimens assessed in the three consecutive periods. Typically, week 21 to week 39 of a year covers the Southern Hemisphere influenza season, while week 40 of a year to week 20 of the following year covers the Northern Hemisphere influenza season. (B) The number of viruses assessed for susceptibility to baloxavir using phenotypic assays and/or sequence-based analysis, by WHO Region for the three consecutive periods. One A(H1N2) virus was included in the baloxavir susceptibility assessment but are not shown in the graph.

**Fig. 5:**
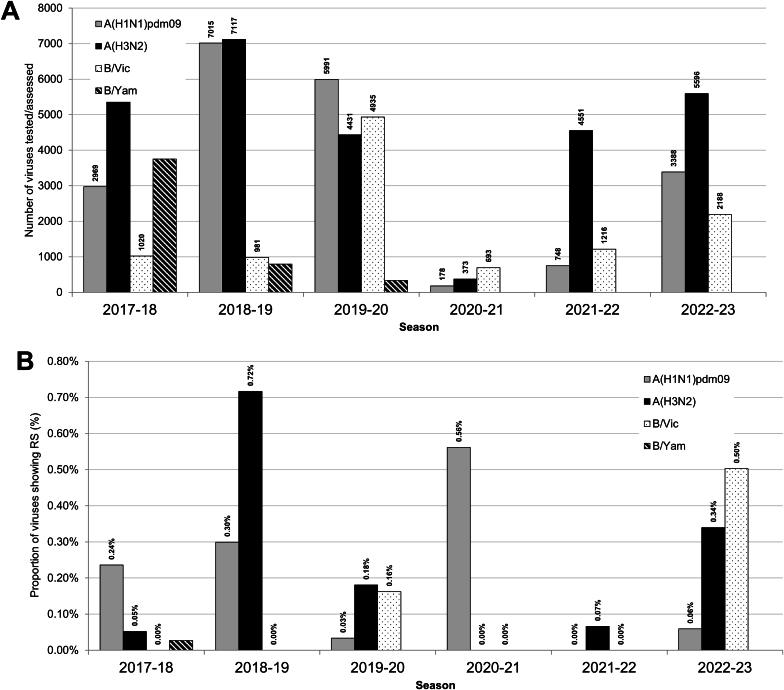
Comparison of baloxavir susceptibility surveillance over six periods for phenotypic and/or sequence-based analysis data generated by WHO CCs. (A) Number of viruses tested. For the 2017–2018 period, baloxavir susceptibility assessment was reported based on sequence-based analysis only. For the 2018–2023 periods, results of phenotypic testing by cell culture-based assays and/or sequence-based analysis were included. (B) The proportion of viruses showing RS to baloxavir over the 2017–2023 periods. Data were compiled from the global studies of viruses during the 2017–2018 (Takashita et al., 2020b), 2018–2020 (Govorkova et al., 2022) and 2020–2023 (current study) periods. For 2017–2018, data from all PA sequences available from public sequence databases; for 2018–2020, data from all PA sequences available from public databases and phenotypic testing data from WHO CCs; for 2020–2023, data from all PA sequences and phenotypic testing data generated by WHO CCs. Viruses characterized by sequence-based analysis were considered RS when the PA contained amino acid substitutions for which the WHO website and/or current testing data showed an >3-fold EC_50_ fold-change compared to baloxavir period median EC_50_ values by subtype/lineage, WHO-CC and method. When a particular PA amino acid substitution listed as RS in the WHO website was phenotypically confirmed NS in the genetic background of current viruses, all with such an amino acid substitution were considered NS (e.g., PA-L28P in A(H3N2) viruses).

During 2020–2021, 2021–2022 and 2022–2023 periods, totals of 1244, 6516 and 11172 viruses, respectively, were assessed for baloxavir susceptibility by five WHO CCs using either phenotypic, sequence-based or both methods (Fig. 4, Fig. 5). These were seasonal influenza A(H1N1)pdm09, A(H3N2) and B/Victoria-lineage viruses (only one A(H1N2) virus in period 2021–2022). The numbers of influenza viruses by type/subtype/lineage collected and assessed for baloxavir susceptibility are shown in Fig. 4, Fig. 5A. Three WHO CCs (Atlanta, Tokyo and London) had tested viruses phenotypically. Phenotypic testing was performed on several viruses representing the different subtypes/lineage in order to calculate the subtype/lineage-specific median EC_50_s. Analysis of PA sequences remained the primary tool for baloxavir susceptibility assessment, as in previous periods (Govorkova et al., 2022). PA sequences were screened for PA markers. Viruses flagged by sequence-based analysis were subjected to phenotypic testing to confirm the drug susceptibility phenotype when isolates were available.

Total numbers of viruses analyzed for baloxavir susceptibility were 1244 [178 A(H1N1)pdm09, 373 A(H3N2), 693 B/Victoria-lineage] for 2020–2021, 6516 [748 A(H1N1)pdm09, 4551 A(H3N2), 1216 B/Victoria-lineage] for 2021–2022 and 11172 [3388 A(H1N1)pdm09, 5596 A(H3N2), 2188 B/Victoria-lineage] for 2022–2023. This represented 10.6% of global influenza detections reported to FluNet for 2020–2021, 1.6% for 2021–2022 and 1.6% for 2022–2023. Significant proportions of the viruses analyzed originated from three WHO regions: WPR with three CCs, 55.2% for 2020–2021, 19.9% for 2021–2022 and 19.2% for 2022–2023; AMR with one CC, 3.9% 2020–2021, 34.1% for 2021–2022 and 40.7% for 2022–2023; and EUR with one CC, 1.4% for 2020–2021, 22.8% for 2021–2022 and 21.4% for 2022–2023. The remaining regions, AFR, EMR and SEAR (all of which do not have a CC), contributed 39.5% of viruses for 2020–2021, 23.2% for 2021–2022 and 18.8% for 2022–2023.

Across all three periods WHO CCs applied sequence-based analysis to virtually all viruses: 1243/1244 (99.9%) in 2020–2021, 6509/6516 (99.9%) in 2021–2022 and 11154/11172 (99.8%) in 2022–2023. Lower proportions were assessed by phenotypic testing: 126/1244 (10.1%) in 2020–2021, 766/6516 (11.8%) in 2021–2022 and 1054/11172 (9.4%) in 2022–2023. The bulk of phenotypic testing was conducted by the Atlanta and Tokyo CCs: 88.1%/11.9% for 2020–2021, 95.2%/4.6% for 2021–2022 and 51.4%/39.0% for 2022–2023.

Overall, combined sequence-based and phenotypic analysis showed the global frequencies of influenza viruses with PA markers was low, being 1/1244 (0.08%), 3/6516 (0.05%) and 32/11172 (0.29%) for the three consecutive periods.

### 3.2 A(H1N1)pdm09 viruses

Only three viruses had PA markers, 1/178 (0.56%), 0/748 (0.00%) and 2/3388 (0.06%) over the three consecutive periods, respectively (Table 3 and S6, Fig. 5). A clinical specimen collected from Togo during 2020–2021 contained PA-E23K substitution (Govorkova et al., 2022; Gubareva et al., 2019; Jones et al., 2022; Takashita et al., 2020a). Patient setting and antiviral treatment status were unknown, and the virus could not be isolated. One virus collected in the United States during 2022–2023 had PA-E199G (both in clinical specimen and isolate) and showed 2.72-fold increase in EC_50_ compared to the subtype-specific median EC_50_. Notably, this virus showed a 4.44-fold increase in EC_50_ compared to a PA sequence-matched control virus, consistent with RS phenotype (Table 3 and S6). One virus from Oman collected during 2022–2023 contained PA-I38V substitution but was not tested phenotypically. Again, patient setting, and antiviral treatment status were unknown.

**Table 3:** WHO CC Data: Virus and patient characteristics for types A and B influenza viruses (n = 36) showing (tested phenotypically) and/or associated (sequence-based analysis) with RS to CENI baloxavir.^a^

Virus (n)	n (sequence-based analysis)	EC_50_ fold-change compared to reference median EC_50_ values^b^	PA substitution^c^		Patient setting	Antiviral treatment	Immunocompromised
		Baloxavir	Virus isolate	Clinical specimen			
**A(H1N1)pdm09** (3 out of 4314)	1 (1)	n/t^d^	E23K	E23K	Unknown	Unknown	Unknown
	1 (1)	2.72^e^	E199G	E199G	Unknown	Unknown	Unknown
	1 (1)	n/t	None^f^	I38V	Unknown	Unknown	Unknown
**A(H3N2)** (22 out of 10520)	5 (2)	**51.54–151**	I38T	I38T (4), I38I/T mix (1)	Community	Yes, baloxavir (3), No (2)	No
	3	**3.49–6.70**	E199G	E199G (3)	Community (3)	No (3)	No (3)
	3 (1)	**8.44–14.59**	I38M (2), Not available (1)	I38M (2), I38I/M mix (1)	Community (1), Unknown (2)	Yes, baloxavir (1), Unknown (2)	No (1), Unknown (2)
	1	**8.72**	I38I/M/T mix	None	Community	Yes, baloxavir	No
	1	**4.69**	Y24C + T357A	Not available	Community	No	No
	1	**3.08**	E199K	E199K	Community	No	No
	1	**5.66**	E23G	E23G	Hospital	Unknown	Unknown
	7	**3.26–8.20**	None	None (1), No data (6)	Unknown	No (2), Unknown (5)	Unknown
**B/Victoria-lineage**	11	**3.09–7.93**	None	No data	Unknown	No (8), Unknown (3)	Unknown
(11 out of 4097)							

a The number (n) of viruses for which data were reported is shown in parentheses if it is less than the number in the ‘n’ column.

b Reduced Susceptibility (RS) fold-change values are displayed underlined and in bold typeface. RS to baloxavir is provisionally defined as >3-fold change in EC_50_ of a test virus compared to the subtype/lineage-specific median EC_50_ values.

c PA amino acid substitutions associated with reduced susceptibility to baloxavir, as listed in the summary table provided by the WHO-AVWG on the WHO website (https://www.who.int/teams/global-influenza-programme/laboratory-network/quality-assurance/antiviral-susceptibility-influenza/polymerase-acidic-protein-inhibitor) are shown.

d n/t: not tested.

e This virus displayed **4.44**-fold change in EC_50_ compared to PA sequence-matched control virus.

f None: No known substitution(s) associated with reduced susceptibility to baloxavir.

### 3.3 A(H3N2) viruses

Only 22 viruses had PA markers and/or showed RS to baloxavir in a phenotypic test, 0/373 (0.00%), 3/4551 (0.07%) and 19/5596 (0.34%) over the three consecutive periods, respectively (Fig. 5). These viruses were collected in 8 countries: Japan (n = 12), Brazil (n = 1), the United States (n = 1), Spain (n = 3), the Netherlands (n = 1), Germany (n = 1), United Arab Emirates (n = 1) and Oman (n = 2) (Table S6). Of these 22 viruses, 11 had PA markers and showed RS phenotype, four had PA markers but were not tested and seven displayed RS phenotype (3.26–8.20-fold) but did not have any PA markers (Table 3 and S6).

**Fig. 6:**
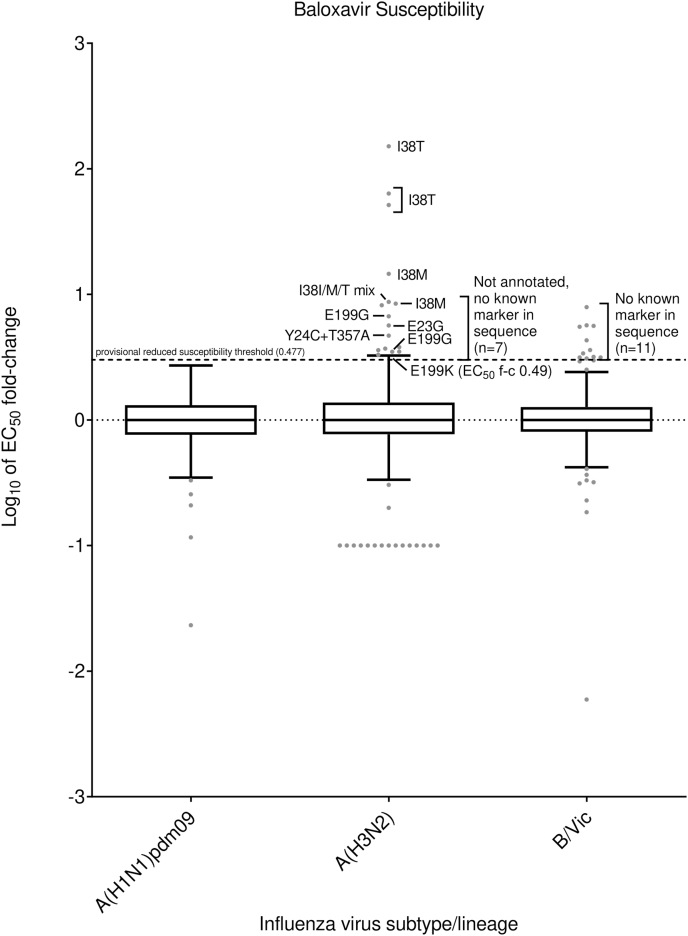
Column-scatter plots of log-transformed 50% effective concentration (EC_50_) fold-change values for baloxavir. The phenotypic susceptibility of influenza viruses to baloxavir was tested by cell culture–based assays, focus-reduction assay (FRA), high-content imaging neutralization test (HINT), or influenza replication inhibition neuraminidase-based assay (IRINA). Overall, 126/1244, 766/6516, and 1054/11172 viruses assessed for baloxavir susceptibility were tested phenotypically for the 2020–2021, 2021–2022 and 2022–2023 periods, respectively. Data are presented by virus subtype or lineage [labelled on the x-axis: A(H1N1)pdm09; A(H3N2); and B/Victoria-lineage] and log-transformed EC_50_ fold changes compared to the period median on the y-axis. The boxes and whiskers are as defined in Fig. 3. An arbitrary cut-off of >3-fold increase in the EC_50_ of a test virus compared to period median EC_50_ was used for reporting viruses with RS to baloxavir. PA amino acid substitutions are shown for viruses displaying a RS phenotype that have been sequenced. Of seven A(H3N2) and eleven B/Victoria-lineage viruses showing an RS phenotype, the PA sequences did not show known markers associated with RS to baloxavir. Amino acid position numbering is specific to type A and B viruses.

Five virus isolates from Japan contained PA-I38T substitution associated with RS to baloxavir. Three of these five viruses were phenotypically tested and showed 51.54- to 151-fold increase in EC_50_ compared to the median EC_50_ value (Table 3 and S6, Fig. 6). Of the five corresponding clinical specimens, four carried PA-I38T and one showed a PA-I38T/I mixture. All five patients were in community settings and three of them had been treated with baloxavir before specimen collection. One virus isolate contained a PA-I38M/T/I mixture, which was not found in the clinical specimen. This virus showed 8.72-fold increased EC_50_ compared to median EC_50_. Two clinical specimens contained PA-I38M (virus could not be isolated from one of the specimens), and patient setting, and antiviral treatment status were unknown (Table 3 and S6, Fig. 6). Another clinical specimen contained PA-I38M/I, while its isolate contained PA-I38M. This virus was collected in a community setting from a baloxavir-treated patient. The two PA-I38M containing viruses were tested phenotypically and showed RS for baloxavir with 8.44- to 14.59-fold increases in EC_50_ compared to median EC_50_. Three viruses from Japan showed PA-E199G, which was present in the clinical specimens; these were taken from a community setting where no antivirals had been used (Table 3 and S6, Fig. 6) (Takashita et al., 2023a). Two viruses were tested phenotypically and showed 3.49- and 6.70-fold increases in EC_50_. One virus carried PA-E199K, also in the clinical specimen taken from a community setting in Japan where no antiviral treatment was administered (Table 3 and S6, Fig. 6). This virus showed a 3.08-fold increased EC_50_ compared to median EC_50_. One virus from the Netherlands possessed PA-E23G, also in the clinical specimen from a hospitalized patient with unknown antiviral treatment (Table 3 and S6, Fig. 6). This virus was tested by both Tokyo and Atlanta WHO CCs, and it showed 4.4–5.66-fold increases in EC_50_ compared to median EC_50_ values in phenotypic assays (FRA, HINT, and IRINA). One virus from Japan contained dual PA-Y24C + PA-T357A substitutions, but the clinical specimen, taken from a community setting where no antiviral treatment was administered, was not available (Table 3 and S6, Fig. 6). This virus showed a 4.69-fold increased EC_50_ compared to median EC_50_. Additionally, seven viruses with no known PA markers showed 3.26- to 8.20-fold increased EC_50_s compared to median EC_50_ (Table 3 and S6, Fig. 6). One virus was tested by Atlanta WHO CC and the other six by London WHO CC. For all seven viruses, the patient settings were unknown, two had not been treated with antivirals and treatment status was unknown for the other five.

### 3.4 B/Victoria-lineage viruses

Only 11 viruses showed RS to baloxavir in a phenotypic test, 0/693 (0.00%), 0/1216 (0.00%) and 11/2188 (0.50%) over the three consecutive periods, respectively (Fig. 5). These viruses were collected in three countries, Belgium (n = 5), Germany (n = 5) and Portugal (n = 1) (Table S6). All viruses had no known PA markers but showed 3.09- to 7.93-fold increases in EC_50_s compared to the reference median EC_50_ value in phenotypic assays conducted by London WHO CC (Table 3 and S6, Fig. 6). For all 11 viruses, no clinical specimen data was available, patient settings were unknown and eight had not received antiviral treatment while treatment status was unknown for the remaining three patients.

### 3.5 Frequency of PA markers associated with RS to baloxavir in sequence databases

We analyzed PA sequences of viruses that were deposited into GISAID, removing duplicate strain designations (preferring sequence data from the original specimen where possible) that were collected during the 2020–2021 (1375 viruses), 2021–2022 (18374 viruses) and 2022–2023 (39927 viruses) periods. According to the strain designations, 1243, 6509 and 11154 sequences from the three consecutive periods belonged to viruses submitted by five WHO CCs for baloxavir susceptibility analysis. For the remaining 132 viruses [32 A(H1N1)pdm09, 62 A(H3N2), 38 B/Victoria-lineage] from 2020–2021, 11865 viruses [768 A(H1N1)pdm09, 10655 A(H3N2), 442 B/Victoria] from 2021–2022 and 28773 viruses [8232 A(H1N1)pdm09, 15111 A(H3N2), 5430 B/Victoria] from 2022–2023, PA sequences were analyzed for the presence of PA markers (Table S7). Mutation calling on sequences downloaded from GISAID was performed using Nextclade (version 3.15.1) (Aksamentov et al., 2021).

For 2020–2021, no viruses showed PA markers. For 2021–2022, six A(H3N2) viruses [0.05% of the total], and for 2022–2023, 16 viruses [0.02% of the total; six A(H1N1)pdm09 and 10 A(H3N2)] were identified with PA markers.

Of the 9032 A(H1N1)pdm09 sequences analyzed, six (0.07%) viruses contained PA markers. Four (66.7%) contained PA-E199G substitution and one virus each (33.3% total) contained PA-E23K or PA-I38V substitutions.

Of the 25828 A(H3N2) sequences analyzed, 16 (0.06%) viruses contained PA markers; seven (43.8%) contained PA-I38T, four (25.0%) viruses contained PA-E199G, two viruses each (25.0% total) viruses contained PA-A36V or PA-I38L, and one virus (6.3%) contained PA-A37T substitution.

Of the 5910 B/Victoria-lineage sequences analyzed, none contained any PA markers (Omoto et al., 2018; Pascua et al., 2022).

Overall, a combined sequence-based and phenotypic analysis demonstrated that the global frequency of all seasonal influenza viruses with RS to baloxavir was low, being 0.07% (1/1376), 0.05% (9/18380) and 0.12% (48/39945) for the three consecutive periods.

## 4 Zoonotic viruses

A total of 111 cases of human infection with swine-lineage [A(H1N1)v, A(H1N2)v, A(H3N2)v subtypes] or avian-lineage [A(H5N1), A(H5N6), A(H5N8), A(H9N2) subtypes] influenza viruses were reported over the three consecutive periods, including 37 cases of infection with highly pathogenic A(H5Nx) viruses (Table S8). Swine influenza zoonotic cases were detected in 11 countries across Europe, North America, South America, Asia and Australia. Avian influenza zoonotic cases were detected in 10 countries across Europe, North America, South America and Asia. The susceptibility of zoonotic influenza viruses to NAIs and baloxavir was assessed based on analysis of NA and PA sequences deposited in GISAID. None of these viruses were tested phenotypically for susceptibility to NAIs or baloxavir. One swine influenza A(H1N1)v virus was identified with NA-H275Y substitution, which is known to confer HRI by oseltamivir and peramivir (NA marker tables) (Baek et al., 2015; Hurt et al., 2009b; Ilyushina et al., 2010; Le et al., 2005; Nguyen et al., 2013). Two highly pathogenic avian influenza viruses belonging to A(H5N1) clade 2.3.2.1c contained NA markers: one contained NA-S247N (Boltz et al., 2010; Nguyen et al., 2023) and the other contained NA-N295S (McKimm-Breschkin et al., 2018; Nguyen et al., 2023). The remaining zoonotic viruses appeared to be susceptible to NAIs, as no NA markers were found in them. None of the viruses contained PA markers. Antiviral treatment history was unavailable for the patients infected with zoonotic influenza viruses.

## 5 Concluding remarks

Seasonal influenza virus circulation in two (2020–2021 and 2021–2022) of the three periods analyzed was markedly lower than in previous periods. It must be noted that the numbers of seasonal influenza viruses assessed for RI/HRI by NAIs or RS to baloxavir in 2022–2023 were over 21-fold and approximately 2-fold higher, respectively, when compared with the 2020–2021 and 2021–2022 periods. The numbers of viruses assessed for RI/HRI by NAIs or RS to baloxavir during the 2018–2019, 2019–2020 periods (Govorkova et al., 2022) and 2022–2023 were similar.

The proportions, 0.09%, 0.12% and 0.23% over the three consecutive periods of seasonal influenza viruses showing RI/HRI by NAIs were lower compared to the pre-COVID-19 2018–2019 (0.5%) and 2019–2020 (0.6%) periods (Govorkova et al., 2022). As in previous periods, the most common marker observed was NA-H275Y in A(H1N1)pdm09 viruses, conferring HRI by oseltamivir and peramivir. In seasons where A(H1N1)pdm09 viruses predominant, higher detection rates of RI/HRI by NAIs tend to occur due to NA-H275Y substitution. For the data presented in this report, in many cases where NA-H275Y variants were detected, associated sample metadata such as patient setting and antiviral treatment status were unavailable; more information is needed in the future to be able to characterize the epidemiology of NA-H275Y in terms of isolated cases of drug resistance versus evidence of human-to-human transmission of these variants. In the case of a pandemic, when vaccines are not yet available, antivirals may be used as first-line countermeasures. Nevertheless, treatment would likely be directed towards high-risk groups rather than massive undirected usage. Moreover, during the 2009 A(H1N1) pandemic, WHO strongly advised against the use of antivirals for prophylactic purposes, even for patients in a high-risk category, due to the increased likelihood of emergence of reduced inhibition to oseltamivir when post-exposure prophylaxis was used. During 2009–2011, emergence of reduced inhibition to oseltamivir was low at <2% (447 out of >27000 viruses tested) (Berera and Zambon, 2013; Hurt et al., 2012; Jones et al., 2023).

A(H3N2) viruses showed lower frequencies of RI/HRI by NAIs than A(H1N1)pdm09 viruses, probably as accumulation of NA markers may come at greater fitness costs than for N1 NAs (Collins et al., 2009). During the 2021–2022 and 2022–2023 periods, the proportions of A(H1N1)pdm09 viruses in circulation was markedly lower compared to those for A(H3N2) and B/Victoria-lineage viruses.

Combined phenotypic and PA sequence-based analysis showed that the global frequency of seasonal influenza viruses with RS to baloxavir or carrying PA markers was low, 0.07%, 0.05% and 0.12% for the three periods, commonly associated with PA-I38T/M/L substitutions. These frequencies were comparable to that reported for 2019–2020 (0.1%), but lower than that reported for 2018–2019 (0.5%) (Govorkova et al., 2022). In Japan, where baloxavir is most used, the frequency was 3.3% in 2022–2023 which was lower than that reported for 2018–2019 (4.5%) (Govorkova et al., 2022). Most of the detected variants (11/16) had PA-I38T/M substitutions, four contained PA-E199G/K and one contained PA-Y24C + PA-T357A. In several cases, no antiviral treatment was administered, suggesting community transmission of viruses bearing PA markers. The limited numbers of influenza viruses isolated in Japan during 2020–2021 and 2021–2022 periods precluded determination of frequencies of RS to baloxavir. However, during 2022–2023, there was a striking difference between Japan and the United States in the detection rates of PA markers. The detection rates in the United States were low in both 2021–2022 (0.06%, 4/6617) and 2022–2023 (0.04%, 4/9318) periods. It has been noted previously that treating children <12 years of age may contribute to the higher rate of RS to baloxavir in Japan (Govorkova et al., 2022). The low detection rate in the United States may be explained by the fact that baloxavir was only approved from August 2022 for treating children (Roche, 2022) and also samples submitted to the United States national surveillance program tend to be collected before treatment is initiated (Gubareva et al., 2019).

Zoonotic viruses isolated from humans (n = 111) in different countries contained N1 NA-H275Y (n = 1), NA-S247N (n = 1) and NA-N295S (n = 1) substitutions associated with RI/HRI by NAIs. None of the viruses showed PA markers. The low detection of markers associated with RI/HRI by NAIs and RS to baloxavir in zoonotic influenza viruses is consistent with pre-COVID-19 pandemic periods (Govorkova et al., 2022).

Combined phenotypic and sequence-based approaches to assess susceptibility to antivirals provide sufficient numbers to be confident in interpretation of the data. However, phenotypic assays require cell culture-propagated virus stocks which may result in artefacts due to amino acid substitutions associated with cell culture adaptation. For example, mixed virus populations can arise and NA substitutions (which can reduce sialidase activity towards sialic acid containing receptors) and PA substitutions can increase the proportion of variants with RI/HRI by NAIs or RS to baloxavir. Sequencing clinical specimens used to obtain virus isolates can improve interpretation of markers associated with RI or RS to antivirals. Moreover, sequence-based analysis allows greater proportions of detected viruses to be included in the dataset as NICs upload large numbers of virus sequences to GISAID while most do not perform phenotypic testing for antiviral susceptibility monitoring. Further, it is the main method used to assess the drug susceptibility of zoonotic influenza viruses as such viruses are often unavailable for phenotypic testing. However, caution must be taken when analyzing sequencing data. It must be noted that markers shown previously to be associated with RI or RS can show no effect in a particular genetic background or have borderline RI or RS phenotypes. Therefore, some studies would report results below the cut-off for RI or RS while others would report them as showing RI or RS. In this report, virus sequences containing markers listed as NI/RI in the WHO-AVWG NA marker table are counted as RI except markers from the current dataset showing NI in phenotypic assays such as NA-N329K/R and NA-S331R in A(H3N2) viruses and NA-K360E in B/Victoria-lineage viruses (previously exhibiting HRI to peramivir). Additionally, markers showing RI/HRI were only counted if previously reported as RI/HRI for that subtype/lineage and if a particular substitution showed an RI/HRI phenotype only in combination with another substitution, then it was not reported as RI/HRI if occurring by itself. This highlights the need to understand the effects of markers for RI in the context of current genetic backgrounds. To gain clearer correlation between identified markers and drug susceptibility phenotype, some laboratories such as Atlanta WHO CC used sequence-matched control viruses (e.g., for PA) or reverse genetics viruses to correlate genetic markers with drug susceptibility phenotypes.

Overall, the frequencies of viruses that showed RI/HRI by NAIs and/or RS to baloxavir was low (<1%) for all three periods reported here regardless of the usage of these antivirals, as was reported in pre-COVID-19 periods (Govorkova et al., 2022). However, the data reported here indicate the need to continue the close monitoring and elucidation of factors contributing to susceptibility of influenza viruses to antiviral drugs.

## CRediT authorship contribution statement

**Saira Hussain:** Writing – original draft, Project administration, Methodology, Investigation, Data curation, Conceptualization. **Adam Meijer:** Writing – review & editing, Project administration, Methodology, Investigation, Formal analysis, Data curation, Conceptualization. **Elena A. Govorkova:** Writing – original draft, Project administration, Methodology, Investigation, Data curation, Conceptualization. **Clyde Dapat:** Formal analysis, Data curation. **Larisa V. Gubareva:** Writing – original draft, Project administration, Methodology, Investigation, Data curation, Conceptualization. **Ian G. Barr:** Supervision, Project administration. **Sook Kwan Brown:** Writing – review & editing, Investigation, Data curation. **Rod S. Daniels:** Writing – review & editing, Project administration, Methodology, Investigation, Data curation, Conceptualization. **Seiichiro Fujisaki:** Writing – review & editing, Investigation. **Monica Galiano:** Project administration, Methodology, Investigation, Data curation, Conceptualization. **Weijuan Huang:** Investigation, Data curation. **Rebecca J. Kondor:** Supervision, Project administration. **Angie Lackenby:** Project administration, Methodology, Investigation, Data curation, Conceptualization. **Nicola Lewis:** Supervision, Project administration. **Janice Lo:** Writing – review & editing, Supervision, Project administration. **Ha T. Nguyen:** Formal analysis, Data curation. **Mira C. Patel:** Writing – review & editing, Methodology, Investigation, Data curation. **Dmitriy Pereyaslov:** Project administration. **Aine Rattigan:** Data curation. **Magdi Samaan:** Writing – review & editing, Supervision, Project administration, Conceptualization. **Dayan Wang:** Supervision, Project administration, Methodology, Investigation, Conceptualization. **Richard J. Webby:** Supervision, Project administration. **Hui-Ling Yen:** Writing – review & editing, Project administration, Methodology, Investigation, Data curation, Conceptualization. **Wenqing Zhang:** Supervision, Project administration. **Emi Takashita:** Writing – review & editing, Project administration, Methodology, Investigation, Data curation, Conceptualization.

## Disclaimer

The authors alone are responsible for the views expressed in this article and they do not necessarily represent the views, decisions, or policies of the institutions with which they are affiliated.

## Declaration of competing interest

The authors declare that they have no known competing financial interests or personal relationships that could have appeared to influence the work reported in this paper.

## Data Availability

Data will be made available on request.
