# Supplementary material for: Global update on the susceptibilities of influenza viruses to neuraminidase inhibitors and the cap-dependent endonuclease inhibitor baloxavir, 2020–2023
Source: Antiviral Res. 2025 Sep;241:106217. doi: 10.1016/j.antiviral.2025.106217 (PMC12391581; doi:10.1016/j.antiviral.2025.106217)
Supplement: Multimedia component 1 [file mmc1.docx]

**Table S1.** Methodology details for Next generation sequencing

| **WHO CCRI** | **RNA extraction** | **RT and Amplicon generation - Primer Scheme** | **RT and Amplicon generation - Enzyme** | **Library prep and Sequencing** | **Analysis software** |
| --- | --- | --- | --- | --- | --- |
| Atlanta | Qiagen - QIAamp viral RNA mini kit | CDC - Uni/Inf primer set | Super-Script III One-Step RT-PCR System with Platinum Taq high fidelity enzyme/Invitrogen | Nextera XT sample preparation kit and Illumina MiSeq | IRMA |
| London | QIAGEN - QIAcube HT | CDC - A/B WGS | QIAGEN OneStep *Ahead*RT-PCR Kit | QIAseq FX DNA Library CDI/UDI | in-house pipeline/scripts https://github.com/Jeremy1805/nf-core-influenzangs.git |
| Melbourne | QIAGEN - QIAcube HT | CDC - A/B WGS | SuperScript IV | Illumina iSeq - DNA Prep | IRMA |
|  | QIAGEN – QIAamp | CDC - A/B HA/NA/MP/PA |  | Nanopore Mk1B – Rapid | Geneious |
|  | QIAGEN - EZ1 | In-house A/B HA/NA/MP/PA (for original clinical specimens) |  | Barcoding kit (RBK) |  |
| Tokyo | Precision System Science/MagLEAD | In-house A/B WGS | Invitrogen/SuperScript III One-Step RT-PCR System with Platinum Taq DNA Polymerase | QIAGEN/QIAseq FX DNA Library Kit | QIAGEN/CLC Genomic Workbench |
|  | Precision System Science/MagDEA Dx SV | In-house A/B per segment (for low-copy viruses) |  |  | IRMA |

**Table S2.** Methodology details for NAI phenotypic assays

| **WHO CCRI** | **Virus pre-titration method** | **Substrate/manufacturer** | **Oseltamivir carboxylate manufacturer** | **Zanamivir manufacturer** | **Peramivir manufacturer** | **Laninamivir manufacturer** | **Fluorometer manufacturer/version** | **Analysis software/version** |
| --- | --- | --- | --- | --- | --- | --- | --- | --- |
| Atlanta | Dilution based on NA activity | NA-Fluor™/Applied Biosystems | Biosynth | Biosynth | Biosynth | Biosynth | BioTek Instruments, Inc./Synergy Neo | Fluoro Dilution Calculations/v3 (CDC developed excel-based calculator for virus dilution), JASPR/v4.X (CDC in-house developed tool for curve fitting) |
| London | Dilution based on NA activity | MUNANA/Biosynth + Merck | Roche | GSK | N/A | N/A | Tecan Infinite F200 Pro | GraFit |
| Melbourne | Dilution based on HA titre | MUNANA/Biosynth | Roche | GSK | BioCryst pharmaceuticals Inc. | Biota | Tecan Infinite F200 Pro | JASPR version 1.2 |
| Tokyo | Dilution based on NA activity | MUNANA/Biosynth | MedChemExpress | MedChemExpress | MedChemExpress | MedChemExpress | Berthold Technologies/TriStar2 LB942 | Labsis Laborsysteme/MikroWin 5.25 GraphPad Software/GraphPad Prism 10 |

| **WHO CCRI** | **Method** | **Cell line** | **Virus pre-titration method** | **Reagents (e.g., substrate or Antibody)/manufacturer** | **Baloxavir manufacturer** | **Plate reader/version** | **Analysis software/version** |
| --- | --- | --- | --- | --- | --- | --- | --- |
| Atlanta | HINT/IRINA | MDCK-SIAT1 | HINT - dilution based on counting immuno-stained infected cell population (~1000 ICP); IRINA - dilution to produce fluorescent signal (RFU) equivalent to 1500 or 750 pmol/well of 4-MU for type A and type B viruses, respectively | HINT - immunostaining with mouse anti-nucleoprotein antibody (International Reagent Resource), followed by incubation with goat anti mouse IgG antibody conjugated to Alexa Fluor-555 (ThermoFisher Scientific), and Hoechst 33,258 dye (AnaSpec Inc.); IRINA - MUNANA (MilliporeSigma) | MedChemExpress | HINT - Nexcelom Celigo® Image cytometer; IRINA - BioTek Cytation 7 | HINT - AFLUT/v6 (CDC in-house developed tool for determining virus dilution and curve fitting); IRINA - CDC IRINA virus dilution calculator/v3 (CDC developed excel-based calculator) and JASPR/v5.X (CDC in-house developed tool for curve fitting) |
| London | MDCK-SIAT1 | NA | Munana | Merck/Biosynth | MedChemExpress | Biotek Cytation 5 /Gen5 software | jaspr5.0 |
| Melbourne | Not done | NA | NA | NA | NA | NA | NA |
| Tokyo | FRA | hCK | Dilution based on Focus Forming Unit | TrueBlue Peroxidase Substrate/SeraCare | Shionogi | Cellular Technology Limited (CTL)/Immunospot S6 Entry M2 | GraphPad Software/GraphPad Prism 10 |

**Table S3.** Methodology details for Baloxavir phenotypic assays

**Table S4.** WHO CC data: Influenza A(H1N1)pdm09, A(H3N2) and B/Victoria-lineage viruses (n=65) showing (tested phenotypically) and/or associated (sequence-based analysis) with RI/HRI by one or more NAIs.

| **#** | **Type/subtype or lineage** | **Strain designation** | **WHO CC** | **Fold-change in IC_50_^a^** | | | | **Substitution in virus isolate^b^** | **Substitution in original specimen^b^** | **Patient setting** | **Antiviral treatment** | **Immuno-compromised** | **Country of specimen collection** | **Date of collection (y/m/d)** |
| --- | --- | --- | --- | --- | --- | --- | --- | --- | --- | --- | --- | --- | --- | --- |
|  |  |  |  | **Oseltamivir** | **Zanamivir** | **Peramivir** | **Laninamivir** |  |  |  |  |  |  |  |
| 1 | A(H1N1)pdm09 | A/Nigeria/5988/2021 | Atlanta | n/t^c^ | n/t | n/t | n/t | Isolate not available | H275Y | Unknown | Unknown | Unknown | Nigeria | 2021-06-03 |
| 2 | A(H1N1)pdm09 | A/Sydney/200/2022 | Melbourne | **2846** | 0.81 | **132** | 1.69 | H275Y | H275Y | Hospital | No | No | Australia | 2022-04-14 |
| 3 | A(H1N1)pdm09 | A/Sydney/202/2022 | Melbourne | **1570** | 0.69 | **130** | 0.83 | H275Y | H275Y | Hospital | No | No | Australia | 2022-04-15 |
| 4 | A(H1N1)pdm09 | A/West Virginia/20/2022 | Atlanta | **1086** | 0.90 | **307** | 1.88 | H275Y | H275Y | Unknown | No | Unknown | the United States | 2022-05-02 |
| 5 | A(H1N1)pdm09 | A/Pennsylvania/37/2022 | Atlanta | **1118** | 1.42 | **386** | 2.33 | H275Y | H275Y | Unknown | No | Unknown | the United States | 2022-05-26 |
| 6 | A(H1N1)pdm09 | A/Pennsylvania/38/2022 | Atlanta | n/t | n/t | n/t | n/t | Isolate not available | H275Y | Unknown | No | Unknown | the United States | 2022-05-29 |
| 7 | A(H1N1)pdm09 | A/India/1491/2022 | Atlanta | **1076** | 1.16 | **251** | 1.81 | H275Y | Original specimen not available | Unknown | Unknown | Unknown | India | 2022-08-06 |
| 8 | A(H1N1)pdm09 | A/Washington/48/2022 | Atlanta | **1066** | 1.21 | **218** | 2.24 | H275Y | H275Y | Unknown | Unknown | Unknown | the United States | 2022-08-27 |
| 9 | A(H1N1)pdm09 | A/India/5/2022 | Melbourne | **665** | 0.98 | **112** | 2.13 | H275Y | H275Y | Unknown | Unknown | Unknown | India | 2022-09-01 |
| 10 | A(H1N1)pdm09 | A/India/4272/2022 | Atlanta | **1359** | 1.11 | **264** | 1.62 | H275Y | Original specimen not available | Unknown | Unknown | Unknown | India | 2022-10-06 |
| 11 | A(H1N1)pdm09 | A/Abu Dhabi/0003849/2022 | Atlanta | **1128** | 1.37 | **264** | 2.43 | H275Y | H275Y | Unknown | Unknown | Unknown | United Arab Emirates | 2022-10-07 |
| 12 | A(H1N1)pdm09 | A/Salamanca/637/2022 | London | **514** | 1.72 | n/t | n/t | H275Y | H275Y | Unknown | Yes, oseltamivir | Yes | Spain | 2022-10-25 |
| 13 | A(H1N1)pdm09 | A/Lebanon/060/2022 | Atlanta | **1020** | 1.00 | **189** | 1.90 | H275Y | H275Y | Unknown | Unknown | Unknown | Lebanon | 2022-11-04 |
| 14 | A(H1N1)pdm09 | A/Salamanca/1221/2022 | London | n/t | n/t | n/t | n/t | H275Y | H275Y | Unknown | Yes, oseltamivir | Yes | Spain | 2022-11-07 |
| 15 | A(H1N1)pdm09 | A/Israel/R10810/2022 | London | **128** | 0.90 | n/t | n/t | H275Y/H (Y75: H25) | Original specimen not available | Unknown | Unknown | Unknown | Israel | 2022-11-08 |
| 16 | A(H1N1)pdm09 | A/California/176/2022 | Atlanta | n/t | n/t | n/t | n/t | H275Y | H275Y | Unknown | Unknown | Unknown | the United States | 2022-11-28 |
| 17 | A(H1N1)pdm09 | A/Victoria/48/2023 | Melbourne | **170** | 1.14 | **13.78** | 1.27 | H275Y | Original specimen not sequenced | Hospital | Unknown | Unknown | Australia | 2023-01-03 |
| 18 | A(H1N1)pdm09 | A/Schweiz/1/2023 | London | **402** | 0.69 | n/t | n/t | H275Y | Original specimen not available | Unknown | Unknown | Unknown | Germany | 2023-01-12 |
| 19 | A(H1N1)pdm09 | A/Netherlands/10294/2023 | London | **601** | 1.92 | n/t | n/t | H275Y | D199G (majority), H275Y (minority) mixture | Unknown | No | Unknown | the Netherlands | 2023-02-16 |
| 20 | A(H1N1)pdm09 | A/Hubei-Dianjun/SWL313/2023 | Beijing | **139** | 1.27 | n/t | n/t | H275Y | Original specimen not available | Unknown | Unknown | Unknown | China | 2023-03-20 |
| 21 | A(H1N1)pdm09 | A/Hubei-Shashi/SWL341/2023 | Beijing | **1222** | 1.05 | n/t | n/t | H275Y | Original specimen not available | Unknown | Unknown | Unknown | China | 2023-03-27 |
| 22 | A(H1N1)pdm09 | A/Tianjin-Nankai/SWL1522/2023 | Beijing | **1448** | 1.09 | n/t | n/t | H275Y | Original specimen not available | Unknown | Unknown | Unknown | China | 2023-04-03 |
| 23 | A(H1N1)pdm09 | A/Anhui-Jinan/SWL1350/2023 | Beijing | **1491** | 1.23 | n/t | n/t | H275Y | Original specimen not available | Unknown | Unknown | Unknown | China | 2023-04-04 |
| 24 | A(H1N1)pdm09 | A/Victoria/380K/2023 | Melbourne | **341** | 1.07 | **96.39** | 2.05 | H275Y/H | Original specimen not sequenced | Hospital | Yes, Zanamivir | Yes | Australia | 2023-04-04 |
| 25 | A(H1N1)pdm09 | A/Shandong-Huaiyin/SWL1304/2023 | Beijing | **774** | 1.40 | n/t | n/t | H275Y | Original specimen not available | Unknown | Unknown | Unknown | China | 2023-04-08 |
| 26 | A(H1N1)pdm09 | A/Victoria/380/2023 | Melbourne | **325** | 0.47 | **178** | 4.88 | H275Y | Original specimen not sequenced | Unknown | Unknown | Unknown | Australia | 2023-04-08 |
| 27 | A(H1N1)pdm09 | A/Victoria/380A/2023 | Melbourne | **930** | 1.12 | **181** | 2.55 | H275Y | Original specimen not sequenced | Unknown | Unknown | Unknown | Australia | 2023-04-08 |
| 28 | A(H1N1)pdm09 | A/Hubei-Xiangcheng/SWL1618/2023 | Beijing | **1289** | 1.20 | n/t | n/t | H275Y | Original specimen not available | Unknown | Unknown | Unknown | China | 2023-04-11 |
| 29 | A(H1N1)pdm09 | A/Victoria/380B/2023 | Melbourne | **974** | 1.21 | **224** | 2.68 | H275Y | H275Y | Unknown | Unknown | Unknown | Australia | 2023-04-12 |
| 30 | A(H1N1)pdm09 | A/Victoria/380C/2023 | Melbourne | **1252** | 1.26 | **321** | 2.39 | H275Y | H275Y | Unknown | Unknown | Unknown | Australia | 2023-04-12 |
| 31 | A(H1N1)pdm09 | A/Victoria/380G/2023 | Melbourne | n/t | n/t | n/t | n/t | H275Y | No data | Unknown | Unknown | Unknown | Australia | 2023-05-04 |
| 32 | A(H1N1)pdm09 | A/Guangxi-Nandan/SWL523/2023 | Beijing | n/t | n/t | n/t | n/t | H275Y | No data | Unknown | Unknown | Unknown | China | 2023-05-05 |
| 33 | A(H1N1)pdm09 | A/Hunan-Yuhu/SWL1529/2023 | Beijing | **776** | 1.11 | n/t | n/t | H275Y | Original specimen not available | Unknown | Unknown | Unknown | China | 2023-05-08 |
| 34 | A(H1N1)pdm09 | A/Victoria/380H/2023 | Melbourne | n/t | n/t | n/t | n/t | H275Y | No data | Unknown | Unknown | Unknown | Australia | 2023-05-11 |
| 35 | A(H1N1)pdm09 | A/Victoria/380J/2023 | Melbourne | **563** | 0.91 | **130** | 1.95 | H275Y | H275Y | Hospital | Unknown | Unknown | Australia | 2023-05-18 |
| 36 | A(H1N1)pdm09 | A/Hunan-Hecheng/SWL1229/2023 | Beijing | 6.34 | **17.24** | n/t | n/t | E119A | Original specimen not available | Unknown | Unknown | Unknown | China | 2023-03-06 |
| 37 | A(H1N1)pdm09 | A/Hubei-Xiling/SWL33/2023 | Beijing | 0.49 | **375** | n/t | n/t | Q136K | Original specimen not available | Unknown | Unknown | Unknown | China | 2023-03-08 |
| 38 | A(H1N1)pdm09 | A/Saitama/1/2022 | Tokyo | n/t | n/t | n/t | n/t | I223T | I223T | Community | No | No | Japan | 2022-08-26 |
| 39 | A(H1N1)pdm09 | A/Perth/54/2023 | Melbourne | n/t | n/t | n/t | n/t | I223T | No data | Unknown | Unknown | Unknown | Australia | 2023-04-27 |
| 40 | A(H1N1)pdm09 | A/Oregon/63/2022 | Atlanta | **11.00** | 1.00 | 1.25 | 1.14 | S247G | S247G | Unknown | Unknown | Unknown | the United States | 2022-11-27 |
| 41 | A(H3N2) | A/Switzerland/09683/2022 | London | n/t | n/t | n/t | n/t | E119V | E119V | Unknown | Unknown | Unknown | Switzerland | 2022-03-30 |
| 42 | A(H3N2) | A/Catalonia/3515398NS/2023 | London | n/t | n/t | n/t | n/t | E119V | No data | Unknown | Unknown | Unknown | Spain | 2023-01-12 |
| 43 | A(H3N2) | A/Tripoli/5646/2021 | London | 1.86 | **10.16** | n/t | n/t | A246V | A246V | Hospital | No | unknown | Lebanon | 2021-09-07 |
| 44 | A(H3N2) | A/Haima/72128470/2021 | London | n/t | n/t | n/t | n/t | K249E | K249E | Unknown | Unknown | Unknown | Oman | 2021-11-10 |
| 45 | B/Vic | B/Fiji/39/2023 | Melbourne | 2.49 | 1.04 | **158** | 0.91 | H134Y | No | Unknown | Unknown | Unknown | Fiji | 2023-02-26 |
| 46 | B/Vic | B/Perth/163/2023 | Melbourne | n/t | n/t | n/t | n/t | G145E | No data | Unknown | Unknown | Unknown | Australia | 2023-03-30 |
| 47 | B/Vic | B/Malaysia/RP2308/2022 | Melbourne | n/t | n/t | n/t | n/t | T146K [culture induced?] | No data | Unknown | Unknown | Unknown | Malaysia | 2022-11-07 |
| 48 | B/Vic | B/Hainan-Xiuying/1611/2021 | Beijing | 3.76 | **13.24** | n/t | n/t | D197E | Original specimen not available | Hospital | Unknown | Unknown | China | 2021-07-22 |
| 49 | B/Vic | B/Nepal/21FL3333/2021 | Tokyo | n/t | n/t | n/t | n/t | Isolate not available | D197E | Unknown | Unknown | Unknown | Nepal | 2021-09-14 |
| 50 | B/Vic | B/Shandong-Huancui/12/2022 | Beijing | n/t | n/t | n/t | n/t | D197E | No data | Unknown | Unknown | Unknown | China | 2022-01-03 |
| 51 | B/Vic | B/Henan-Xigong/1515/2021 | Beijing | **5.00** | **8.52** | n/t | n/t | D197N | Original specimen not available | Hospital | Unknown | Unknown | China | 2021-11-15 |
| 52 | B/Vic | B/Anhui-Yingjiang/12512/2021 | Beijing | n/t | n/t | n/t | n/t | D197N | No data | Unknown | Unknown | Unknown | China | 2021-11-25 |
| 53 | B/Vic | B/Beijing-Huairou/1418/2022 | Beijing | n/t | n/t | n/t | n/t | D197N | No data | Unknown | Unknown | Unknown | China | 2022-03-13 |
| 54 | B/Vic | B/Romania/550346/2023 | London | n/t | n/t | n/t | n/t | D197N | No data | Unknown | Unknown | Unknown | Romania | 2023-04-12 |
| 55 | B/Vic | B/St. Petersburg/RII-06/2022 | London | 1.09 | **5.76** | n/t | n/t | I262M [I263M, if accounting for ins73L] | No data | Unknown | Unknown | Unknown | Russian Federation | 2022-04-27 |
| 56 | B/Vic | B/St. Petersburg/RII-15/2022 | London | 1.76 | **6.01** | n/t | n/t | I262M [I263M, if accounting for ins73L] | No data | Unknown | Unknown | Unknown | Russian Federation | 2022-05-26 |
| 57 | B/Vic | B/Guangxi-Qinbei/32/2021 | Beijing | 0.06 | **5.79** | n/t | n/t | H273Q | Original specimen not available | Hospital | Unknown | Unknown | China | 2021-05-08 |
| 58 | B/Vic | B/Amazonas/2023-019853-IEC/2023 | Atlanta | n/t | n/t | n/t | n/t | G407S | No data | Unknown | Unknown | Unknown | Brazil | 2023-05-11 |
| 59 | B/Vic | B/Catalonia/3511044NS/2022 | London | 0.77 | **8.86** | n/t | n/t | None | None | Unknown | No | Unknown | Spain | 2022-12-04 |
| 60 | B/Vic | B/Catalonia/2279261NS/2023 | London | 1.07 | **5.54** | n/t | n/t | None | None | Unknown | Yes, unknown | Unknown | Spain | 2023-01-03 |
| 61 | B/Vic | B/Netherlands/10195/2023 | London | 1.80 | **6.98** | n/t | n/t | None | None | Unknown | No | Unknown | the Netherlands | 2023-01-31 |
| 62 | B/Vic | B/Netherlands/10258/2023 | London | 1.26 | **6.26** | n/t | n/t | None | None | Unknown | No | Unknown | the Netherlands | 2023-02-09 |
| 63 | B/Vic | B/Bulgaria/1485/2023 | London | 2.19 | **6.25** | n/t | n/t | S244P | No data | Unknown | Unknown | Unknown | Bulgaria | 2023-02-14 |
| 64 | B/Vic | B/Slovenia/732/2023 | London | 1.85 | **6.13** | n/t | n/t | None | No data | Unknown | Unknown | Unknown | Slovenia | 2023-03-06 |
| 65 | B/Vic | B/Bulgaria/1896/2023 | London | 1.22 | **5.25** | n/t | n/t | None | None | Unknown | Unknown | Unknown | Bulgaria | 2023-03-27 |

^a^Reduced inhibition (RI) and highly reduced inhibition (HRI) fold-change values are displayed in bold typeface. For type A viruses, normal inhibition (NI) is a <10-fold increase in the NAI IC_50_; RI is a 10- to 100-fold increase; and HRI is a >100-fold increase, for type B viruses, NI is a <5-fold increase in the NAI IC_50_; RI is a 5- to 50-fold increase; and HRI is a >50-fold increase (WHO, 2012).

^b^NA amino acid position numbering is A-subtype or B-type specific. NA amino acid substitutions associated with RI/HRI, as listed in the summary table provided by the WHO-AVWG on the WHO website (<https://www.who.int/teams/global-influenza-programme/laboratory-network/quality-assurance/antiviral-susceptibility-influenza/neuraminidase-inhibitor>) are shown.

^c^n/t: not tested.

**Table S5.** Influenza type A and B viruses carrying NA substitutions associated with RI/HRI by NAIs based on analysis of sequences from non-WHO CC data available in GISAID.^a^

| ***#*** | **Type/subtype or lineage** | **Strain designation** | **Submitting laboratory** | **NA substitution^b^** | **Passage details/history^c^** | **NA GISAID Acc. No.** | **Country of specimen collection** | **Season** |
| --- | --- | --- | --- | --- | --- | --- | --- | --- |
| 1 | A(H1N1)pdm09 | A/Saint-Petersburg/RII-125/2023 | WHO National Influenza Centre Russian Federation | D199G | Cell | EPI2590680 | Russian Federation | 2022-2023 |
| 2 | A(H1N1)pdm09 | A/Saint-Petersburg/RII-8841S/2023 | WHO National Influenza Centre Russian Federation | D199G | Original | EPI2325456 | Russian Federation | 2022-2023 |
| 3 | A(H1N1)pdm09 | A/Netherlands/10370-1/2020 | National Institute for Public Health and the Environment (RIVM) | H275Y | Original | EPI1838444 | Netherlands | 2020-2021 |
| 4 | A(H1N1)pdm09 | A/Baltimore/JH-674/2024 | Johns Hopkins School of Medicine | H275Y | Cell | EPI3325465 | the United States | 2021-2022 |
| 5 | A(H1N1)pdm09 | A/Netherlands/00393/2022 | Erasmus Medical Center | H275Y | Original | EPI2010572 | Netherlands | 2021-2022 |
| 6 | A(H1N1)pdm09 | A/Abu Dhabi/UAE/0007992/2022 | Sheikh Khalifa Medical City (SKMC) | H275Y | Cell | EPI2682372 | United Arab Emirates | 2022-2023 |
| 7 | A(H1N1)pdm09 | A/Abu Dhabi/UAE/0008378/2022 | Sheikh Khalifa Medical City (SKMC) | H275Y | Cell | EPI2922773 | United Arab Emirates | 2022-2023 |
| 8 | A(H1N1)pdm09 | A/Arkhangelsk/CRIE/444/2022 | Central Research Institute of Epidemiology | H275Y | Cell | EPI2296090 | Russian Federation | 2022-2023 |
| 9 | A(H1N1)pdm09 | A/Baltimore/JH-152/2023 | Johns Hopkins School of Medicine | H275Y | Cell | EPI3074333 | the United States | 2022-2023 |
| 10 | A(H1N1)pdm09 | A/Beijing/CY00004/2023 | China CDC | H275Y | Cell | EPI2664492 | China | 2022-2023 |
| 11 | A(H1N1)pdm09 | A/England/225260275/2022 | UK Health Security Agency - Colindale | H275Y | Original | EPI2384099 | United Kingdom | 2022-2023 |
| 12 | A(H1N1)pdm09 | A/France/ARA-HCL023014596001/2023 | CNR Virus des Infections Respiratoires, France SUD | H275Y | Cell | EPI2492824 | France | 2022-2023 |
| 13 | A(H1N1)pdm09 | A/France/ARA-HCL023032643101/2023 | CNR Virus des Infections Respiratoires, France SUD | H275Y | Cell | EPI2567831 | France | 2022-2023 |
| 14 | A(H1N1)pdm09 | A/India/Pun-NIVARI56/Sep2022 | ICMR-National Institute of Virology | H275Y | Cell | EPI2413894 | India | 2022-2023 |
| 15 | A(H1N1)pdm09 | A/India/Pun-NIVCOV2224272/Jun2022 | ICMR-National Institute of Virology | H275Y | Cell | EPI2413950 | India | 2022-2023 |
| 16 | A(H1N1)pdm09 | A/India/Pun-NIVSARI1491/Jun2022 | ICMR-National Institute of Virology | H275Y | Cell | EPI2413926 | India | 2022-2023 |
| 17 | A(H1N1)pdm09 | A/Lebanon/GIHSN-HCL023019592501/2022 | CNR Virus des Infections Respiratoires, France SUD | H275Y | Cell | EPI2386692 | Lebanon | 2022-2023 |
| 18 | A(H1N1)pdm09 | A/Lebanon/GIHSN-HCL023019598301/2022 | CNR Virus des Infections Respiratoires, France SUD | H275Y | Cell | EPI2386701 | Lebanon | 2022-2023 |
| 19 | A(H1N1)pdm09 | A/Llay/8431/2022 | Public Health Wales Microbiology Cardiff | H275Y | Cell | EPI2244008 | United Kingdom | 2022-2023 |
| 20 | A(H1N1)pdm09 | A/Norway/05139/2023 | Norwegian Institute of Public Health | H275Y | Original | EPI2529589 | Norway | 2022-2023 |
| 21 | A(H1N1)pdm09 | A/Novosibirsk/RII-7.271S/2022 | WHO National Influenza Centre Russian Federation | H275Y | Original | EPI2324251 | Russian Federation | 2022-2023 |
| 22 | A(H1N1)pdm09 | A/Oklahoma/13801/2022 | U.S. Air Force School of Aerospace Medicine | H275Y | Original | EPI2298580 | the United States | 2022-2023 |
| 23 | A(H1N1)pdm09 | A/PaisVasco/4350/2022 | Instituto de Salud Carlos III | H275Y | Original | EPI2273970 | Spain | 2022-2023 |
| 24 | A(H1N1)pdm09 | A/Romania/545719/2023 | Cantacuzino Institute | H275Y | Original | EPI2547047 | Romania | 2022-2023 |
| 25 | A(H1N1)pdm09 | A/Romania/626/2023 | National Institute of Infectious Diseases-Prof. Dr. Matei Bals | H275Y | Cell | EPI2462245 | Romania | 2022-2023 |
| 26 | A(H1N1)pdm09 | A/Romania/84/2023 | National Institute of Infectious Diseases-Prof. Dr. Matei Bals | H275Y | Cell | EPI2462003 | Romania | 2022-2023 |
| 27 | A(H1N1)pdm09 | A/Saint-Petersburg/RII-4917S/2022 | WHO National Influenza Centre Russian Federation | H275Y | Original | EPI2263352 | Russian Federation | 2022-2023 |
| 28 | A(H1N1)pdm09 | A/Santiago/34412/2023 | Instituto de Salud Pública de Chile | H275Y | Cell | EPI2715242 | Chile | 2022-2023 |
| 29 | A(H1N1)pdm09 | A/Saransk/RII-MH128677S/2022 | WHO National Influenza Centre Russian Federation | H275Y | Original | EPI2556248 | Russian Federation | 2022-2023 |
| 30 | A(H1N1)pdm09 | A/Saransk/RII-MH128724S/2023 | WHO National Influenza Centre Russian Federation | H275Y | Original | EPI2556448 | Russian Federation | 2022-2023 |
| 31 | A(H1N1)pdm09 | A/Staraya Russa/RII-MH132845S/2023 | WHO National Influenza Centre Russian Federation | H275Y | Original | EPI2591237 | Russian Federation | 2022-2023 |
| 32 | A(H1N1)pdm09 | A/Staraya Russa/RII-MH132854S/2023 | WHO National Influenza Centre Russian Federation | H275Y | Original | EPI2591242 | Russian Federation | 2022-2023 |
| 33 | A(H1N1)pdm09 | A/Surat Thani/P1451/2023 | Thai National Influenza Center | H275Y | Original | EPI2588995 | Thailand | 2022-2023 |
| 34 | A(H1N1)pdm09 | A/Velikiy Novgorod/RII-MH132665S/2023 | WHO National Influenza Centre Russian Federation | H275Y | Original | EPI2575788 | Russian Federation | 2022-2023 |
| 35 | A(H1N1)pdm09 | A/Saint-Petersburg/RII-5118S/2022 | WHO National Influenza Centre Russian Federation | I223K | Original | EPI2264798 | Russian Federation | 2022-2023 |
| 36 | A(H1N1)pdm09 | A/Abu Dhabi/UAE/0007623/2022 | Sheikh Khalifa Medical City (SKMC) | I223M | Cell | EPI2642522 | United Arab Emirates | 2022-2023 |
| 37 | A(H1N1)pdm09 | A/Castilla La Mancha/931/2023 | Instituto de Salud Carlos III | I223M | Original | EPI2520150 | Spain | 2022-2023 |
| 38 | A(H1N1)pdm09 | A/Castilla La Mancha/934/2023 | Instituto de Salud Carlos III | I223R | Original | EPI2520166 | Spain | 2022-2023 |
| 39 | A(H1N1)pdm09 | A/France/ARA-HCL023031721301/2023 | CNR Virus des Infections Respiratoires, France SUD | I223R | Cell | EPI2567722 | France | 2022-2023 |
| 40 | A(H1N1)pdm09 | A/Saint-Petersburg/RII-52/2022 | WHO National Influenza Centre Russian Federation | I223R | Cell | EPI2558759 | Russian Federation | 2022-2023 |
| 41 | A(H1N1)pdm09 | A/Amazonas/2023-010775-IEC/2023 | Evandro Chagas Institute | I223T | Cell | EPI2607434 | Brazil | 2022-2023 |
| 42 | A(H1N1)pdm09 | A/Luxembourg/LNS1093736/2023 | Laboratoire National de Santé | I223T | Cell | EPI2684350 | Luxembourg | 2022-2023 |
| 43 | A(H1N1)pdm09 | A/Michigan/HFHSL361450519/2023 | University of Michigan | I223T | Original | EPI2624659 | the United States | 2022-2023 |
| 44 | A(H1N1)pdm09 | A/Switzerland/28184/2023 | Swiss National Reference Centre for Influenza | I223T | Original | EPI2686372 | Switzerland | 2022-2023 |
| 45 | A(H1N1)pdm09 | A/Astrakhan/RII-MH129970S/2023 | WHO National Influenza Centre Russian Federation | I223V+H275Y | Original | EPI2560398 | Russian Federation | 2022-2023 |
| 46 | A(H1N1)pdm09 | A/Baltimore/JH-97/2023 | Johns Hopkins School of Medicine | N295S | Cell | EPI3073569 | the United States | 2021-2022 |
| 47 | A(H1N1)pdm09 | A/Saint-Petersburg/RII-157/2022 | WHO National Influenza Centre Russian Federation | Q136R | Cell | EPI2559287 | Russian Federation | 2022-2023 |
| 48 | A(H1N1)pdm09 | A/Cheboksary/293-t2V/2023 | State Research Center of Virology and Biotechnology (VECTOR) | R152K | Cell | EPI2582747 | Russian Federation | 2022-2023 |
| 49 | A(H1N1)pdm09 | A/Moscow/32/2022 | WHO National Influenza Centre Russian Federation | S247G | Cell | EPI2322918 | Russian Federation | 2022-2023 |
| 50 | A(H3N2) | A/Shanghai/FX1518C2/2022 | NA | D151G | Cell | EPI3588138 | China | 2022-2023 |
| 51 | A(H3N2) | A/Jiangsu-wuxi/0801/2022 | Wuxi Center for Disease Control and Prevention | D151G | Cell | EPI2543756 | China | 2022-2023 |
| 52 | A(H3N2) | A/Jiangsu-wuxi/0858/2022 | Wuxi Center for Disease Control and Prevention | D151G | Cell | EPI2543748 | China | 2022-2023 |
| 53 | A(H3N2) | A/Switzerland/9650/2022 | Swiss National Reference Centre for Influenza | E119V | Original | EPI2121378 | Switzerland | 2021-2022 |
| 54 | A(H3N2) | A/Catalonia/NSVH101970147/2022 | Hospital Universitari Vall d'Hebron | E119V | Original | EPI2247674 | Spain | 2022-2023 |
| 55 | A(H3N2) | A/England/230420251/2023 | UK Health Security Agency - Colindale | E119V | Original | EPI2469517 | United Kingdom | 2022-2023 |
| 56 | A(H3N2) | A/United Kingdom/GSTT_IAV_A1.4/2023 | NA | E119V | Cell | EPI2541437 | United Kingdom | 2022-2023 |
| 57 | A(H3N2) | A/Paris/01327/2023 | Institut Pasteur | E119V | Original | EPI2414854 | France | 2022-2023 |
| 58 | A(H3N2) | A/England/230720061/2023 | UK Health Security Agency - Colindale | E119V | Original | EPI2538941 | United Kingdom | 2022-2023 |
| 59 | A(H3N2) | A/Baltimore/JH-140/2021 | Johns Hopkins School of Medicine | E276D | Cell | EPI2040779 | the United States | 2021-2022 |
| 60 | A(H3N2) | A/Netherlands/00010/2022 | Erasmus Medical Center | K249E | Original | EPI1967357 | Netherlands | 2021-2022 |
| 61 | A(H3N2) | A/England/220760408/2022 | UK Health Security Agency - Colindale | K249E | Original | EPI1995795 | United Kingdom | 2021-2022 |
| 62 | A(H3N2) | A/Baltimore/JH-0481/2022 | Johns Hopkins School of Medicine | K249E | Cell | EPI2216479 | the United States | 2022-2023 |
| 63 | A(H3N2) | A/Baltimore/JH-0530/2022 | Johns Hopkins School of Medicine | K249E | Cell | EPI2216783 | the United States | 2022-2023 |
| 64 | A(H3N2) | A/Baltimore/JH-0498/2022 | Johns Hopkins School of Medicine | K249E | Cell | EPI2216599 | the United States | 2022-2023 |
| 65 | A(H3N2) | A/New York/RVTNL3333274854327/2022 | University of Michigan | K249E | Original | EPI2509472 | the United States | 2022-2023 |
| 66 | A(H3N2) | A/Michigan/UOM10049213332/2023 | University of Michigan | K249E | Original | EPI2593574 | the United States | 2022-2023 |
| 67 | A(H3N2) | A/Norway/00863/2022 | Norwegian Institute of Public Health | K249E | Original | EPI2381656 | Norway | 2022-2023 |
| 68 | A(H3N2) | A/Sao Paulo/IAL/C8803/2022 | Instituto Adolfo Lutz | N142S | Original | EPI1983965 | Brazil | 2021-2022 |
| 69 | A(H3N2) | A/Canada/Nova Scotia/32077/2022 | QEII health Sciences Centre | N245Y | Cell | EPI2850583 | Canada | 2022-2023 |
| 70 | A(H3N2) | A/Paris/05459/2023 | Institut Pasteur | R292K | Original | EPI2525993 | France | 2022-2023 |
| 71 | B/Victoria | B/Nordrhein-Westfalen/43/2023 | Robert Koch Institute Nationales Referenzzentrum für Influenza | D197E | Original | EPI2612182 | Germany | 2022-2023 |
| 72 | B/Victoria | B/Shandongrencheng/1127/2022 | Jining Center for Disease Control and Prevention | D197N | Cell | EPI2528129 | China | 2021-2022 |
| 73 | B/Victoria | B/Khabarovsk/215/2022 | WHO National Influenza Centre Russian Federation | D197N | Cell | EPI2573341 | Russian Federation | 2022-2023 |
| 74 | B/Victoria | B/Rio de Janeiro/26730/2022 | Instituto Oswaldo Cruz FIOCRUZ - Laboratory of Respiratory Viruses and Measles (LVRS) | D197N | Original | EPI2328051 | Brazil | 2022-2023 |
| 75 | B/Victoria | B/Austria/1598591/2023 | Medical University Vienna | D432N | Original | EPI2500548 | Austria | 2022-2023 |
| 76 | B/Victoria | B/Norway/05951/2023 | Norwegian Institute of Public Health | D432N | Original | EPI2573812 | Norway | 2022-2023 |
| 77 | B/Victoria | B/Kenya/GIHSN-HCL023181312201/2023 | CNR Virus des Infections Respiratoires, France SUD | G243S | Original | EPI2863293 | Kenya | 2022-2023 |
| 78 | B/Victoria | B/Luxembourg/LNS8679822/2023 | Laboratoire National de Santé | G407S | Cell | EPI2683836 | Luxembourg | 2022-2023 |
| 79 | B/Victoria | B/Nakhon Phanom/P4101/2022 | Thai National Influenza Center | G407S | Original | EPI2304888 | Thailand | 2022-2023 |
| 80 | B/Victoria | B/Austria/1584780/2023 | Medical University Vienna | H273Y | Original | EPI2350052 | Austria | 2022-2023 |
| 81 | B/Victoria | B/Galicia/870/2023 | Instituto de Salud Carlos III | H273Y | Cell | EPI2519538 | Spain | 2022-2023 |
| 82 | B/Victoria | B/Peru/UPCH_3871/2023 | Universidad Peruana Cayetano Heredia | I221L | Cell | EPI2580635 | Peru | 2022-2023 |
| 83 | B/Victoria | B/Santiago/13527/2023 | Instituto de Salud Pública de Chile | I221T | Cell | EPI2714926 | Chile | 2022-2023 |
| 84 | B/Victoria | B/Catalonia/NSAV198271989/2023 | Hospital Universitari Vall d'Hebron | I221V | Original | EPI2538744 | Spain | 2022-2023 |
| 85 | B/Victoria | B/Singapore/SAR0028/2022 | Ministry of Health, Singapore | N151S | Original | EPI2092066 | Singapore | 2021-2022 |
| 86 | B/Victoria | B/Singapore/SAR1224/2022 | Ministry of Health, Singapore | N151S | Original | EPI2092114 | Singapore | 2021-2022 |
| 87 | B/Victoria | B/Singapore/SAR94214/2021 | Ministry of Health, Singapore | N151S | Original | EPI1956520 | Singapore | 2021-2022 |
| 88 | B/Victoria | B/Singapore/SAR94800/2021 | Ministry of Health, Singapore | N151S | Original | EPI1956552 | Singapore | 2021-2022 |

^a^GISAID: Global Initiative on Sharing All Influenza Data; RI: reduced inhibition; HRI: highly reduced inhibition. NA sequences of influenza viruses collected during 2020-2021, 2021-2022 and 2022-2023 periods were downloaded from GISAID. To ensure accuracy, the sequences were curated to remove duplicate sequences of individual viruses and preference was given to the sequence from the original clinical specimen, if available. Totals of 166, 12869 and 27956 NA sequences for the three periods were analyzed for NA amino acid substitutions.

^b^NA amino acid position numbering is A-subtype or B-type specific. NA amino acid substitutions associated with RI/HRI, as listed in the summary table provided by the WHO-AVWG on the WHO website (<https://www.who.int/teams/global-influenza-programme/laboratory-network/quality-assurance/antiviral-susceptibility-influenza/neuraminidase-inhibitor>) are shown. Substitutions shown in the WHO summary table as NI/RI are included in table S2. However, substitutions which were previously associated with RI or HRI but in the 2020-2023 period (viruses with different genetic backbones) consistently showed NI in NA inhibition assays were excluded from Table S2. These substitutions were NA-N329K/R or NA-S331R in A(H3N2) and NA-K360E in B/Victoria lineage viruses.

^c^Passage as shown in GISAID.

**Table S6.** WHO CC Data: Influenza A(H1N1)pdm09, A(H3N2), and B/Victoria-lineage viruses (n=36) showing (tested phenotypically) and/or associated (sequence-based analysis) with RS to CENI baloxavir.

|  | **Type/subtype or lineage** | **Strain designation** | **WHO CC** | **Fold change in EC_50_^a^** | **PA substitution in virus isolate^b^** | **PA substitution in original specimen^b^** | **Patient setting** | **Antiviral treatment** | **Immuno-compromised** | **Country of specimen collection** | **Date of collection (y/m/d)** |
| --- | --- | --- | --- | --- | --- | --- | --- | --- | --- | --- | --- |
| 1 | A(H1N1)pdm09 | A/Togo/0263/2021 | Atlanta | n/t^c^ | Isolate not available | E23K | Unknown | Unknown | Unknown | Togo | 2021-03-05 |
| 2 | A(H1N1)pdm09 | A/Oregon/67/2022 | Atlanta | 2.72^d^ | E199G | E199G | Unknown | Unknown | Unknown | the United States | 2022-11-29 |
| 3 | A(H1N1)pdm09 | A/Salalah/5239001/2023 | London | n/t | None | I38V | Unknown | Unknown | Unknown | Oman | 2023-03-26 |
| 4 | A(H3N2) | A/Netherlands/10253/2022 | London/Tokyo/  Atlanta^e^ | **5.66** | E23G | E23G; by submitting lab | Hospital | Unknown | Unknown | the Netherlands | 2022-03-06 |
| 5 | A(H3N2) | A/Kanagawa/IC2236/2023 | Tokyo | **8.72** | I38M/T/I mix | No | Community | Baloxavir | No | Japan | 2023-04-11 |
| 6 | A(H3N2) | A/Brazil/8979/2021 | Atlanta | n/t | Isolate not available | I38M | Unknown | Unknown | Unknown | Brazil | 2021-12-21 |
| 7 | A(H3N2) | A/Illinois/01/2022 | Atlanta | **8.44** | I38M | I38M | Unknown | Unknown | Unknown | the United States | 2022-01-25 |
| 8 | A(H3N2) | A/Shizuoka/3/2023 | Tokyo | **14.59** | I38M | I38M/I mix | Community | Baloxavir | No | Japan | 2023-01-12 |
| 9 | A(H3N2) | A/Yamagata/18/2023 | Tokyo | **151** | I38T | I38T | Community | No | No | Japan | 2023-02-04 |
| 10 | A(H3N2) | A/Hiroshima/42/2023 | Tokyo | **63.60** | I38T | I38T | Community | No | No | Japan | 2023-03-09 |
| 11 | A(H3N2) | A/Kanagawa/IC2235/2023 | Tokyo | **51.54** | I38T | I38T/I mix | Community | Baloxavir | No | Japan | 2023-04-06 |
| 12 | A(H3N2) | A/Miyazaki/68/2023 | Tokyo | n/t | I38T | I38T | Community | Baloxavir | No | Japan | 2023-05-19 |
| 13 | A(H3N2) | A/Miyazaki/73/2023 | Tokyo | n/t | I38T | I38T | Community | Baloxavir | No | Japan | 2023-05-20 |
| 14 | A(H3N2) | A/Nara/10/2023 | Tokyo | n/t | E199G | E199G | Community | No | No | Japan | 2023-02-20 |
| 15 | A(H3N2) | A/Nara/12/2023 | Tokyo | **6.70** | E199G | E199G | Community | No | No | Japan | 2023-02-21 |
| 16 | A(H3N2) | A/Nara/14/2023 | Tokyo | **3.49** | E199G | E199G | Community | No | No | Japan | 2023-03-03 |
| 17 | A(H3N2) | A/Shizuoka/31/2023 | Tokyo | **3.08** | E199K | E199K | Community | No | No | Japan | 2023-05-07 |
| 18 | A(H3N2) | A/Kobe/22067/2022 | Tokyo | **4.69** | Y24C+T357A | Original specimen not available | Community | No | No | Japan | 2022-12-12 |
| 19 | A(H3N2) | A/Abu Dhabi/1722389/2022 | Atlanta | **3.26** | None | None | Unknown | Unknown | Unknown | United Arab Emirates | 2022-06-21 |
| 20 | A(H3N2) | A/Ibra/72211258/2022 | London | **3.30** | None | No data | Unknown | Unknown | Unknown | Oman | 2022-10-02 |
| 21 | A(H3N2) | A/Burgos/26/2022 | London | **3.80** | None | No data | Unknown | No | Unknown | Spain | 2022-10-04 |
| 22 | A(H3N2) | A/Ibra/72211780/2022 | London | **8.20** | None | No data | Unknown | Unknown | Unknown | Oman | 2022-10-20 |
| 23 | A(H3N2) | A/Salamanca/36/2022 | London | **3.60** | None | No data | Unknown | Unknown | Unknown | Spain | 2022-11-02 |
| 24 | A(H3N2) | A/Salamanca/39/2022 | London | **3.50** | None | No data | Unknown | Unknown | Unknown | Spain | 2022-12-07 |
| 25 | A(H3N2) | A/Bayern/6/2023 | London | **3.70** | None | No data | Unknown | No | Unknown | Germany | 2023-01-26 |
| 26 | B/Vic | B/Belgium/S1989/2022 | London | **3.18** | None | No data | Unknown | Unknown | Unknown | Belgium | 2022-09-30 |
| 27 | B/Vic | B/Berlin/7/2022 | London | **3.60** | None | No data | Unknown | Unknown | Unknown | Germany | 2022-11-09 |
| 28 | B/Vic | B/Belgium/G0334/2022 | London | **3.15** | None | No data | Unknown | No | Unknown | Belgium | 2022-12-05 |
| 29 | B/Vic | B/Belgium/G0370/2022 | London | **5.68** | None | No data | Unknown | No | Unknown | Belgium | 2022-12-12 |
| 30 | B/Vic | B/Belgium/G0384/2022 | London | **5.62** | None | No data | Unknown | No | Unknown | Belgium | 2022-12-13 |
| 31 | B/Vic | B/Belgium/S2629/2022 | London | **3.09** | None | No data | Unknown | No | Unknown | Belgium | 2022-12-13 |
| 32 | B/Vic | B/Lisboa/11/2022 | London | **3.39** | None | No data | Unknown | Unknown | Unknown | Portugal | 2022-12-30 |
| 33 | B/Vic | B/Hessen/9/2023 | London | **4.31** | None | No data | Unknown | No | Unknown | Germany | 2023-02-13 |
| 34 | B/Vic | B/Berlin/18/2023 | London | **7.93** | None | No data | Unknown | No | Unknown | Germany | 2023-02-14 |
| 35 | B/Vic | B/Nordrhein-Westfalen/9/2023 | London | **3.15** | None | No data | Unknown | No | Unknown | Germany | 2023-02-16 |
| 36 | B/Vic | B/Nordrhein-Westfalen/13/2023 | London | **5.52** | None | No data | Unknown | No | Unknown | Germany | 2023-02-17 |

^a^Fold change in baloxavir susceptibility compared to subtype/lineage-specific medians. Fold change values >3, the provisional threshold for reduced susceptibility are displayed in bold typeface.

^b^PA amino acid substitutions potentially associated with reduced baloxavir susceptibility, as listed in the summary table provided by the WHO-AVWG on the WHO website (<https://www.who.int/teams/global-influenza-programme/laboratory-network/quality-assurance/antiviral-susceptibility-influenza/polymerase-acidic-protein-inhibitor>) are shown.

^c^n/t: not tested.

^d^This virus displayed **4.44**-fold change in EC_50_ compared to PA sequence-matched control virus.

^e^Phenotypically tested by Tokyo WHO CC (fold-change shown in Table) and Atlanta WHO CC (compared to PA-sequence matched virus HINT fold-change **4.4** and IRINA **4.8**).

**Table S7.** Influenza type A and B viruses carrying PA substitutions of concern associated with RS to CENI baloxavir based on analysis of sequences from non-WHO CC data available in GISAID.^a^

| **#** | **Type/subtype or lineage** | **Strain designation** | **Submitting laboratory** | **PA substitution^b^** | **Passage details/history^c^** | **PA GISAID Acc. No.** | **Country of specimen collection** | **Season** |
| --- | --- | --- | --- | --- | --- | --- | --- | --- |
| 1 | A(H1N1)pdm09 | A/Baltimore/JH-876/2023 | Johns Hopkins School of Medicine | E199G | Cell | EPI3075509 | the United States | 2022-2023 |
| 2 | A(H1N1)pdm09 | A/France/ARA-HCL022216396001/2022 | CNR Virus des Infections Respiratoires, France SUD | E199G | Cell | EPI2491779 | France | 2022-2023 |
| 3 | A(H1N1)pdm09 | A/Khabarovsk/135/2022 | WHO National Influenza Centre Russian Federation | E199G | Cell | EPI2590293 | Russian Federation | 2022-2023 |
| 4 | A(H1N1)pdm09 | A/Michigan/UOM10048476259/2022 | University of Michigan | E199G | Original | EPI2311099 | the United States | 2022-2023 |
| 5 | A(H1N1)pdm09 | A/Michigan/UOM10049022432/2022 | University of Michigan | E23K | Original | EPI2672956 | the United States | 2022-2023 |
| 6 | A(H1N1)pdm09 | A/Paracatu/311504015/2023 | Ezequiel Dias Foundation (FUNED) | I38V | Original | EPI2952792 | Brazil | 2022-2023 |
| 7 | A(H3N2) | A/Netherlands/01162/2022 | Erasmus Medical Center | A36V | Original | EPI2129601 | Netherlands | 2021-2022 |
| 8 | A(H3N2) | A/Netherlands/11604/2022 | National Institute for Public Health and the Environment (RIVM) | A36V | Original | EPI2055588 | Netherlands | 2021-2022 |
| 9 | A(H3N2) | A/Texas/12888/2022 | U.S. Air Force School of Aerospace Medicine | A37T | Original | EPI2054977 | the United States | 2021-2022 |
| 10 | A(H3N2) | A/England/220880277/2022 | UK Health Security Agency - Colindale | E199G | Original | EPI2073796 | United Kingdom | 2021-2022 |
| 11 | A(H3N2) | A/Michigan/UOM10045351706/2022 | University of Michigan | E199G | Original | EPI2094087 | the United States | 2021-2022 |
| 12 | A(H3N2) | A/Belgium/H0002/2023 | SCIENSANO | E199G | Original | EPI2879924 | Belgium | 2022-2023 |
| 13 | A(H3N2) | A/Belgium/H0005/2023 | SCIENSANO | E199G | Original | EPI2879932 | Belgium | 2022-2023 |
| 14 | A(H3N2) | A/Catalonia/NSVH531042678/2022 | Hospital Universitari Vall d'Hebron | I38L | Original | EPI2052681 | Spain | 2022-2023 |
| 15 | A(H3N2) | A/Egypt/HT220391/2022 | Ain Shams University, Faculty of Science | I38L | Cell | EPI3605761 | Egypt | 2022-2023 |
| 16 | A(H3N2) | A/Texas/12816/2022 | U.S. Air Force School of Aerospace Medicine | I38T | Original | EPI2054434 | the United States | 2021-2022 |
| 17 | A(H3N2) | A/Catalonia/NSVH198259859/2022 | Hospital Universitari Vall d'Hebron | I38T | Original | EPI2222297 | Spain | 2022-2023 |
| 18 | A(H3N2) | A/Nagasaki/22FS102/2023 | Niigata University (DPH) | I38T | Original | EPI2529325 | Japan | 2022-2023 |
| 19 | A(H3N2) | A/Niigata/22FS109_2/2023 | Niigata University (DPH) | I38T | Original | EPI2529349 | Japan | 2022-2023 |
| 20 | A(H3N2) | A/Niigata/22FS137_2/2023 | Niigata University (DPH) | I38T | Original | EPI2529365 | Japan | 2022-2023 |
| 21 | A(H3N2) | A/Salamanca/283/2022 | Valladolid National Influenza Centre | I38T | Cell | EPI2932793 | Spain | 2022-2023 |
| 22 | A(H3N2) | A/Tokyo/22FS161_2/2022 | Niigata University (DPH) | I38T | Original | EPI3139880 | Japan | 2022-2023 |

^a^GISAID: Global Initiative on Sharing All Influenza Data; PA sequences of influenza viruses collected during 2020-2021, 2021-2022, and 2022-2023 periods were downloaded from GISAID. To ensure accuracy, the sequences were curated to remove duplicate sequences for individual viruses and preference was given to the sequence from the original clinical specimen, if available. Totals of 97, 11195 and 22719 PA sequences for the three consecutive periods were analyzed for PA amino acid substitutions.

^b^PA amino acid substitutions potentially associated with reduced baloxavir susceptibility as listed in the summary table provided by the WHO-AVWG on the WHO website (<https://www.who.int/teams/global-influenza-programme/laboratory-network/quality-assurance/antiviral-susceptibility-influenza/polymerase-acidic-protein-inhibitor>) are shown. Substitutions shown in the WHO summary table as conferring normal susceptibility or reduced susceptibility (NS/RS) are included in this table. During the 2020-2023 period and previous 2018-2020 period (Govorkova et al., 2022), A(H3N2) virus isolates carrying PA-L28P tested in phenotypic assay consistently displayed normal susceptibility to baloxavir. Therefore, A(H3N2) viruses with PA-L28P were not included in this table.

^c^Passage as shown in GISAID.

**Table S8**. Zoonotic influenza type A viruses (n=111) carrying NA and PA substitutions associated with reduced susceptibility to NAIs and CENI baloxavir.

| **Influenza A virus subtype (clade)** | **n** | **Geographical area of virus isolation (n)** | **Amino acid substitutions associated with reduced antiviral susceptibility (n)^a^** | | **GISAID accession number for identified substitutions** | |
| --- | --- | --- | --- | --- | --- | --- |
|  |  |  | **NA substitution** | **PA substitution** | **NA gene** | **PA gene** |
| ***Swine influenza*** | | | | | | |
| H1N1v (1A3.3.2pdm09; 1A3.3.3 Gamma; 6B.1A.1; 1C2.1pdm09; 1C2.2; 1C2.3; 1C2.6) | 25 | Denmark (3), Germany (4), Netherlands (1), Spain (1), USA (8), Brazil (1), China (7) | H275Y (1)^b^ | None^c^ | EPI1838444 | N/A^d^ |
| H1N2v (1A.1.1 Alpha; 1A3.3.2; 1B.2.1 Delta2; 1C2.4; 1C2.5) | 21 | France (1), Netherlands (1), Canada (2), USA (15), Brazil (1), Taiwan (1) | None | None | N/A | N/A |
| H3N2v | 10 | Canada (1), USA (8), Australia (1) | None | None | N/A | N/A |
| ***Avian influenza*** | | | | | | |
| H5N1 (2.3.2.1c) (HPAI)^e^ | 7 | United Kingdom (1), USA (1), Cambodia (3), India (1), Laos (1) | S247N (1),  N295S (1) | None | EPI1842224,  EPI2116744 | N/A |
| H5N1 (2.3.4.4b) (HPAI) | 11 | United Kingdom (5), Spain (2), USA (1), Ecuador (1), Chile (1), China (1) | None | None | N/A | N/A |
| H5N6 (2.3.4.4b; 2.3.4.4.h) (HPAI) | 18 | China (18) | None | None | N/A | N/A |
| H5N8 (2.3.4.4b) (HPAI) | 1 | Russia (1) | None | None | N/A | N/A |
| H9N2 | 18 | Cambodia (2), China (16) | None | None | N/A | N/A |

^a^Amino acid position numbering is A-subtype specific. NA amino acid substitutions associated with RI/HRI, as listed in the summary table provided by the WHO-AVWG on the WHO website (<https://www.who.int/teams/global-influenza-programme/laboratory-network/quality-assurance/antiviral-susceptibility-influenza/neuraminidase-inhibitor>) are shown.

^b^Patient was treated with oseltamivir.

^c^None: no substitutions previously reported to be associated with RI/HRI by NAIs or RS to CENI baloxavir were identified.

^d^N/A: not applicable.

^e^HPAI: Highly Pathogenic Avian Influenza virus.
